# Investigating the miRNA-mRNA interactome of human trabecular meshwork cells treated with TGF-β1 provides insights into the pathogenesis of pseudoexfoliation glaucoma

**DOI:** 10.1371/journal.pone.0318125

**Published:** 2025-01-30

**Authors:** Anton W. Roodnat, Chelsey Doyle, Breedge Callaghan, Karen Lester, Megan Henry, Carl Sheridan, Declan J. McKenna, Colin E. Willoughby, Sarah D. Atkinson

**Affiliations:** 1 Biomedical Sciences Research Institute, Centre for Genomic Medicine, Ulster University, Coleraine, Northern Ireland, United Kingdom; 2 Department of Eye and Vision Science, Institute of Life Course and Medical Sciences, University of Liverpool, Liverpool, United Kingdom; Royan Institute for Stem Cell Biology and Technology, ISLAMIC REPUBLIC OF IRAN

## Abstract

Pseudoexfoliation glaucoma is a severe form of secondary open angle glaucoma and is associated with activation of the TGF-β pathway by TGF-β1. MicroRNAs (miRNAs) are small non-coding RNA species that are involved in regulation of mRNA expression and translation. To investigate what glaucomatous changes occur in the trabecular meshwork and how these changes may be regulated by miRNAs, we performed a bioinformatics analysis resulting in a miRNA-mRNA interactome. Primary human trabecular meshwork cells originating from normal donors were treated with TGF-β1 at 5 ng/mL for 24h; total RNA was extracted followed by RNA-Seq and miRNA-Seq. For both mRNA and miRNA species, differential expression was determined using a bioinformatics pipeline consisting of FastQC, STAR, FeatureCounts, edgeR (for miRNA) and DESeq2 (for mRNA). Putative mRNA-miRNA interactions between differentially expressed mRNA and miRNA species were determined using interaction databases miRWalk, miRTarBase, TarBase and TargetScan. To classify mRNA species by function and pathway, gene enrichment was performed using Enrichr. The resulting miRNA-mRNA interactome consisted of 1202 interactions. Some highly connected microRNAs were hsa-let-7e-5p, hsa-miR-20a-5p, hsa-miR-122-5p, and hsa-miR-29c-3p. Most differentially expressed genes were indicated to be regulated by miRNAs. The sub-interactomes of genes involved in specific pseudoexfoliation glaucoma related enrichment terms such as oxidative stress, unfolded protein response, signal molecules and ECM remodelling were determined. This is the first study to present a genome-wide microRNA-mRNA regulatory network for human trabecular meshwork cells treated with TGF-β1 and may serve to generate unbiased hypotheses about regulatory functions and mRNA targets of miRNAs in pseudoexfoliation glaucoma and may help to develop miRNA-based therapeutics.

## Introduction

Pseudoexfoliation glaucoma (XFG) is a severe form of secondary open angle glaucoma which develops from pseudoexfoliation syndrome (XFS). The prevalence of XFS has been estimated to be 60 million people and in half this population pseudoexfoliation glaucoma (XFG) will eventually develop [1]. The aetiology of pseudoexfoliation glaucoma is not fully understood but it is associated with the production and release of exfoliation material (XFM) in the anterior segment of the eye which is deposited in the trabecular meshwork (TM) and other structures within the anterior segment [2]. Deposition of XFM in the TM is believed to contribute to the increased intraocular pressure (IOP) observed in XFG by blocking the outflow facility. Important components of XFM are amongst others *LOXL1*, fibrillin, latent TGF-β binding proteins and elastin [3]. Conventional glaucoma therapies lower IOP pharmacologically or surgically [4] but cellular and molecular changes in the TM in XFG remained unchecked. Understanding the mechanisms underlying the regulation of gene and protein expression in the TM in XFG would provide a foundation for developing gene-based therapies for this clinically aggressive glaucoma [5,6]. The pathogenesis of XFS and XFG is multifactorial and is associated with single nucleotide polymorphisms of *LOXL1* [7,8] and other genes [2] but also with ageing [1], UV exposure [1], folate intake [9], hyperhomocysteinaemia [10], oxidative stress [11], dysfunctional autophagy [12] and dysregulated retinoic acid signalling [13]. Furthermore, XFG is associated with increased concentration of TGF-β1, both in active form in the aqueous humour and in latent form which is bound to the extracellular matrix [14]. TGF-β1 is an extracellular signal molecule involved in several cellular processes such as growth, extracellular matrix remodelling, oxidative stress, cell migration and apoptosis and is associated with wound-healing and fibrosis [15]. Treatment of normal TM cells with TGF-β1 induces changes in biological processes that show many similarities with changes observed in the TM in XFG such as production of XFM components, activation of the unfolded protein response, changes in the antioxidant system and fibrotic ECM remodelling [16].

MicroRNAs (miRNAs) are small noncoding RNA species that bind to specific mRNA sequences, usually in the 3’ untranslated region, and by doing so regulate gene expression by increased mRNA degradation or by reducing translation of the target mRNA. MiRNA regulation of gene expression is not very gene-specific, but a miRNA rather regulates a group (module) of genes and expression of one gene may be regulated by several miRNAs simultaneously [17]. Several miRNAs have been found to be differentially expressed in ocular tissues from XFG patients compared to normal tissue [2,18,19]. Furthermore, when normal TM cells are treated with TGF-β1 this induces changes in the expression of miRNAs [20] and there is a significant overlap with changes in miRNA expression observed in XFG. Previously several specific miRNA-gene interactions have been studied in ocular tissues [21–23] but these studies only reveal a small fraction of a vast interactive miRNA-gene network at the genome level. A representation of this genome-wide network in which all putative miRNA-gene interactions are shown for a certain condition is called a miRNA-gene interactome. Such genome-wide interactomes have been reported for other conditions such as osteoarthritis [24–26], ventricular hypertrophy [27] and cancer [28–31] but not for the trabecular meshwork. Therefore, the main goal of this work was to construct a miRNA::mRNA interactome for TM cells treated with TGF-β1 using our previously published miRNA [20] and mRNA [16] datasets, to determine how differentially expressed genes and altered biological processes may be regulated by miRNAs on a genome-wide level to investigate the role of miRNAs in XFG pathology. This interactome may help to understand the pathophysiology of XFG and could be used to identify interesting candidates for miRNA-based therapeutics.

## Materials and methods

### mRNA and miRNA sequencing

Primary normal human trabecular meshwork (TM) tissue was harvested from donor eyes originating from four different donors (n = 4) obtained from the Liverpool Research Eye Bank in the period 01/06/2018–01/01/2019.

Medical history for the donor eyes was unknown, however, no donors had previous ocular surgery or a known glaucoma diagnosis. Donor eyes were excluded if the maximum post-mortem time exceeded 48 h. More detailed donor information can be found in our previous publications [16,20] and the associated Short Read Archive BioProjects PRJNA1099874 and PRJNA882267 as mentioned in those publications. TM cells from four different donors were cultured followed by either TGF-β1 treatment or no treatment (i.e. controls) resulting in a paired experiment. The RNA concentration was measured using the NanoDrop 2000 (Thermofisher Scientific, Horsham, UK) and was higher than 50 ng/uL for miRNA and higher than 20 ng/uL for mRNA for all samples. RNA quality was determined using the Bioanalyzer 2100 (Agilent Technologies, Stockport, UK) and measured RIN was higher than 8 for miRNA and higher than 7 for mRNA. RNA extracted from all eight cell cultures was subjected to both regular RNA sequencing and small RNA sequencing. More details concerning the sequencing process can be found in our previous publications [16,20]. Subsequent data processing resulted in differentially expressed mRNA species and associated functional enrichment [16] and differentially expressed miRNA species [20] as reported previously by our group.

### miRNA-mRNA interaction database

A combined miRNA-mRNA interaction database (or more loosely miRNA-gene interaction database) was created by combining multiple online available databases into one merged database in order to align gene and miRNA symbols, accession codes and the interaction evidence presented by the databases (e.g. experimental versus predicted interactions). Both predicted miRNA::mRNA interactions from TargetScan 8.0 (accessed 01/10/2022) [32] and miRWalk 2.0 (accessed 30/09/2022) [33] together with experimentally confirmed interactions from Tarbase v8 (accessed 25/01/2021) [34] and miRTarBase 8.0 (accessed 20/01/2021) [35] were added to establish a combined database of miRNA::mRNA interactions using an R script. In addition, the combined database mirDIP (accessed 30/10/2022) [36] was called from an R script and results were added to this ‘in-house’ combined miRNA::mRNA interaction database. From each database, interactions were selected using lists of expressed mRNAs and miRNAs obtained from our RNA-Seq and miRNA-Seq experiments as described above. Next it was attempted to estimate a probability of interaction in the form of a p-value using the information available in each online interaction database. In miRTarBase, the strength of evidence was used (strong: p = 0.01, weaker: p = 0.1); in TargetScan, the p-value was estimated using the context-score value. In TarBase, p = 0.01 was assigned to interactions obtained by Western Blot, qPCR or luciferase assays and p = 0.1 to other interactions. Furthermore, all TarBase interactions obtained via indirect evidence were filtered out and excluded. In miRWalk, binding probability was used as a measure of p-value (so p = 1 –binding probability). In mirDIP only interactions with an interaction score of at least ‘high’ were utilised and the ‘confidence score’ was mapped to a p-value. After combining the interactions from these databases, the final p-value was determined by taking the lowest (i.e. best) value of individual p-values per database. The rationale for this method is that if database A provides strong evidence (e.g. p = 0.01) and another database B provides weaker evidence (e.g. p = 0.1) then the information from database A is most likely to be correct and hence p = 0.01. After combining, the total miRNA::mRNA interaction database for human TM cells consisted of 3,711,158 interactions between 2757 unique miRNAs and 13784 unique genes.

### Creation of a miRNA-mRNA interactome

**Fig 1:**
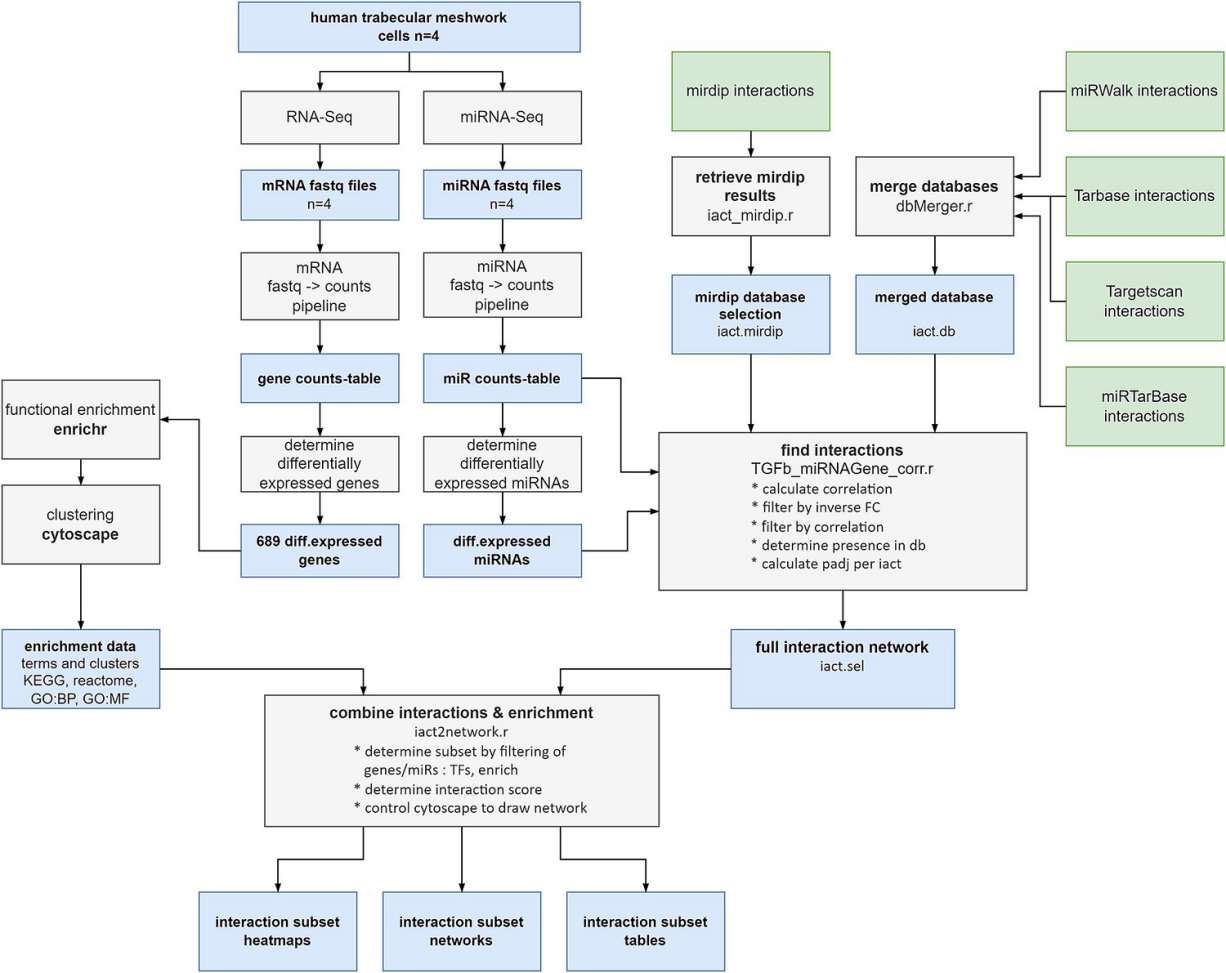
Bioinformatics flow. Shown is the bioinformatics flow that has been used to create the miRNA-gene interactome in which blue boxes indicate data objects, grey boxes indicate processing steps and green boxes indicate external databases. Human trabecular meshwork cells are sequenced to determine both mRNA (genes) and miRNA species. Furthermore, an interaction database is compiled using publicly available interaction databases. This results in a full interaction network which can be utilised to extract subsets of the complete network for certain enrichment themes. Such a subset can be presented as heatmap, cytoscape network or table.

Using sets of differentially expressed (FDR<0.05, |log_2_FC|>0.26) miRNAs and mRNAs together with the combined miRNA-mRNA database, a set of miRNA-mRNA interactions was created as depicted in Fig 1. This set of interactions was subjected to the following filters:

1. Differential expression of the regulating miRNA should be in the opposite direction to the differential expression of the regulated mRNA so if the expression of a regulating miRNA is increased then the expression of the regulated mRNA should be decreased and vice versa (Inverse Expression Correlation).
2. If a gene is indicated to be regulated by multiple miRNA species, then the miRNAs that have an expression level that is at least 8 orders lower than the expression level of the miRNA with the highest expression level (as expressed in log_2_CPM) are filtered out. This is a novel step intended to weed out less important miRNA regulators. The value 8 (equivalent to a 256x lower expression) was chosen based on the observation that the range of expression of a set of miRNAs regulating a particular gene can be in the order of 12 (when expressed as log_2_CPM) so 8 seems to be a fair threshold value.
3. Both participants in a miRNA::mRNA interaction should be significantly differentially expressed when corrected for multiple observations, i.e. the false discovery rate (FDR) should be <0.05 and the absolute value of the log2 of fold-change |log_2_FC| >0.26.

This FDR can be calculated in two ways: the simplest approach is to only use miRNA and mRNA species of which the FDR<0.05; this is the standard interactome similar to other studies [24].

Instead of correcting for multiple hypothesis testing by calculating the FDR for mRNA and miRNA separately, which would also correct for non-interacting miRNA-mRNA combinations, another option is to calculate the p-value per interaction based on the p-values of the regulating miRNA and regulated mRNA and then determine the FDR for the set of interactions. Assuming it is certain that a miRNA will interact with a certain mRNA and we require that both participants should be differentially expressed due to TGF-β1 treatment then the p-value for a certain interaction reflects the probability that either miRNA or mRNA is not differentially expressed and can be calculated as follows:

$$p_{interaction}=p_{mRNA}*(1‐p_{miRNA})+p_{miRNA}*(1‐p_{mRNA})+p_{mRNA}*p_{miRNA}\approx p_{mRNA}+p_{miRNA}$$ (1)

So, for example, if the p-value of a regulating miRNA is 0.04 and that of the regulated mRNA is 0.05 then the probability that at least one of these is in fact not differentially expressed is approximately 0.09. Using this method, an enhanced list of interactions and their p-values was obtained. Using this set, the (Benjamini-Hochberg) FDR was calculated per interaction and only interactions with FDR<0.05 were selected.

In summary the total set of interactions consists of standard interactions in which both miRNA and regulated gene are differentially expressed when corrected for multiple testing, so FDR<0.05 for both gene and miRNA, and the enhanced set of interactions in which the interaction is significant (FDR<0.05) when corrected for multiple testing. Because the number of interactions is different from the number of genes or miRNAs, the correction for multiple testing will be different.

At this point, we have arrived at a ‘Boolean’ interactome which shows that there may or not may be an interaction but there is no information about the interaction strength. To get some sense of the regulatory strength of an interaction, an interaction score S was estimated that contains miRNA molarity (in the form of CPM), Pearson correlation in expression between miRNA and mRNA and the evidence presented in the combined interaction database as follows:

S=evidence.exper+standard.iact+2*abs(cor)+0.2*mir.molarity (2)

in which evidence.exper = 1 if the considered interaction has been reported to be confirmed experimentally in a database, not just predicted. Parameter ‘standard.iact’ represents if the interaction originates from the standard rather than the enhanced interactome, in which case the value will be 1, otherwise 0. Parameter ‘abs(cor)’ represents the absolute value of the Pearson correlation between expression of miR and mRNA for this interaction and ‘mir.molarity’ is the molarity of the miRNA expressed as log_2_CPM.

The theoretical maximum value of this interaction score is about 7 for those interactions that (1) have been proven experimentally (2) both gene and miRNA have FDR<0.05 (3) correlation = -1 (4) high miRNA molarity, in practice limited to 15 or so. It should be noted that the Pearson correlation per sample could be calculated in our analysis because both RNA-Seq and miRNA-Seq were performed on the same samples. More details and considerations with respect to the use of these filters can be found in the discussion. The code to reproduce this analysis has been made available on github, see section ‘Data Availability’.

### Enrichment analysis of interactions

To investigate regulation of subsets of mRNAs (genes) by miRNAs, functional enrichment analysis of differentially expressed genes was performed as described in [16]. In short, Enrichr was used to arrive at a set of enrichment terms from KEGG, Reactome and GO biological processes and molecular functions which were subsequently clustered based on gene overlap. Using these enrichment results and the gene-miRNA interactome it can be determined what miRNAs may play a role in a certain enrichment cluster by determining what cluster-genes are part of the interactome and by what miRNA species they are regulated. This analysis results in a matrix of enrichment clusters versus differentially expressed miRNA species. Another level of detail can be obtained by analysing gene-miRNA interactions per enrichment cluster resulting in an interaction matrix of genes versus miRNAs for one particular enrichment cluster of interest. To determine what differentially expressed genes in our dataset are transcription factors, gene list GO:0000981 called “DNA-binding transcription factor activity, RNA polymerase II-specific” was used. In addition to subsets that have been obtained after enrichment, it is also interesting to investigate what genes may be regulated by a set of miRNA species (such as the miR-29 family).

The code to reproduce this analysis has been made available on github, see section ‘Data Availability’.

### Determining miRNA regulation of biological processes

To estimate what biological processes a certain differentially expressed miRNA may be regulating, a matrix of miRNAs versus functional enrichment clusters was created in which each matrix entry represents the number of genes regulated by a certain miRNA for a certain functional enrichment cluster divided by the total number of miRNA-regulated genes in that cluster so each matrix entry must be between 0.0 (this miRNA does not regulate any genes in this enrichment cluster) and 1.0 (all regulated genes in this cluster are also regulated by this miRNA).

### Analysis of miRNA—transcription factor loops

By combining interactions in which a miRNA is regulating a transcription factor (TF) and interactions in which a TF regulates the expression of a miRNA, feedback loops may be detected. To determine what genes may be transcription factors, GO gene list “RNA polymerase II cis-regulatory region sequence-specific DNA binding” (GO:0000978) was used. To determine which miRNAs may be regulated by these TFs, TF–miRNA interactions in which TFs regulate miRNA expression were obtained from miRNet (accessed 29/04/2023) [37]. By combining both types of interactions, it becomes possible to detect double negative miRNA—| TF—| miRNA feedback loops. In such a loop, an upregulated TF should have a repressive effect on miRNA transcription and this repression will cause higher expression of the miRNA-controlled TF.

### Ethics statement

The study was conducted in accordance with the Declaration of Helsinki and ethical approval was obtained from the local ethics review board of the University of Liverpool (RETH000833) in addition to national ethical approval from the UK National Research Ethics Service Committee North West—Haydock (IRAS ID 119105; REC reference 13/NW/0061, extended by IRAS ID 239185; REC reference 18/NW/0136). Written consent from relatives of the eye donors was obtained by the Liverpool Research Eye Bank.

## Results

### Differentially expressed mRNA and miRNA species in response to TGF-β1 stimulation

As reported earlier by our group, 689 mRNA species were found to be differentially expressed with a false discovery rate (FDR) < 0.05 in primary human TM cells after treatment with TGF-β1 (5ng/mL) for 24 hrs when compared to untreated controls [16]. Functional annotation of the set of differentially expressed genes set resulted in 219 enrichment terms originating from KEGG, Reactome, Gene Ontology (GO) biological processes and molecular functions. Using gene set overlap analysis several enrichment terms could be clustered into 53 functional clusters [16]. Furthermore, in the same samples, 107 miRNAs were found to be differentially expressed with p<0.05 [20] of which 52 miRNAs were differentially expressed when corrected for multiple testing i.e. FDR<0.05.

### miRNA-mRNA interactome for TGF-β1 stimulated human TM cells

**Fig 2:**
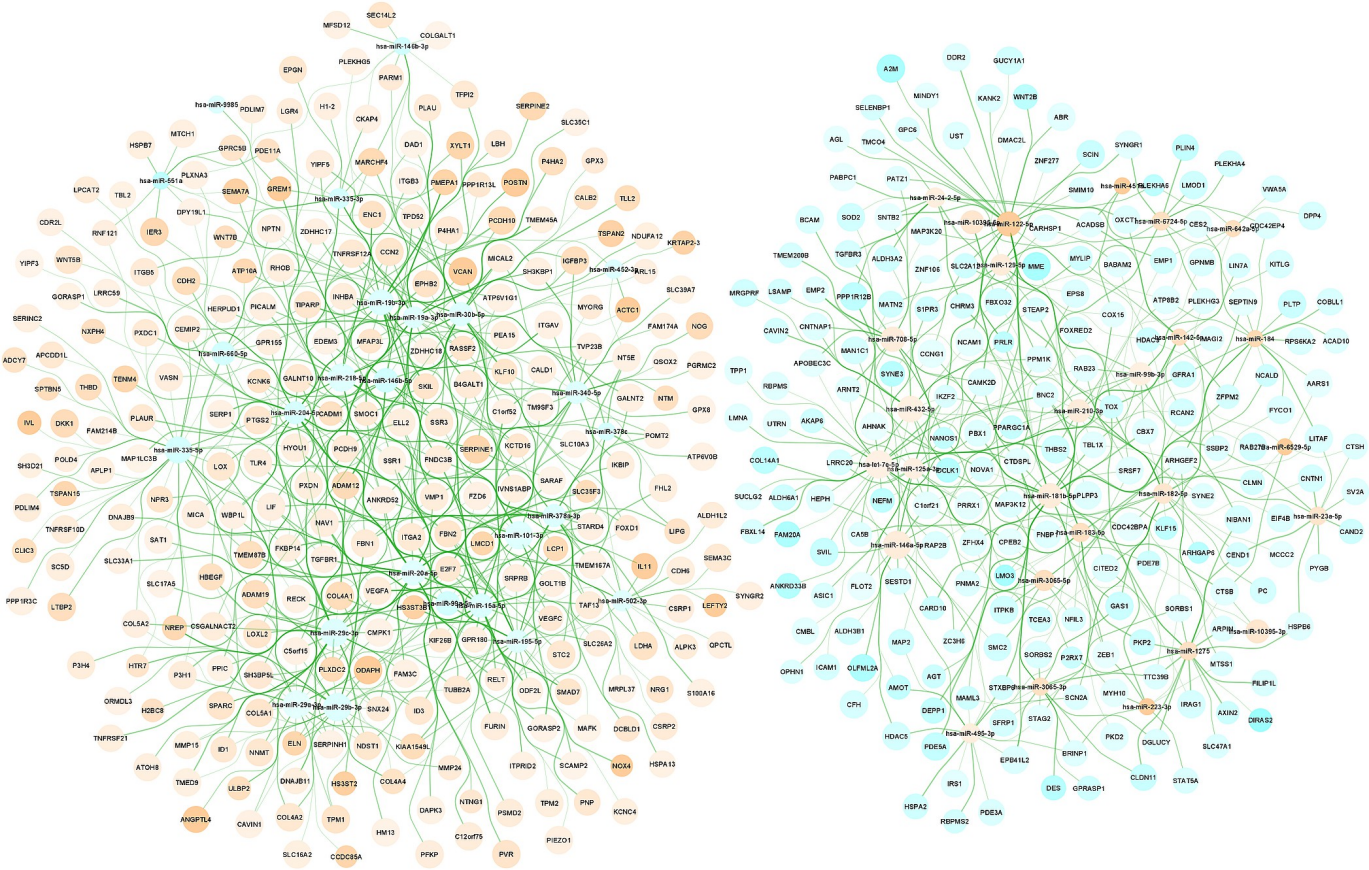
Standard miRNA-gene interactome of human trabecular meshwork cells stimulated by TGF-β1. The node colour indicates if the RNA species (miRNA or mRNA) is upregulated (orange) or downregulated (blue) and the colour intensity has been mapped to log_2_FC. The node size has been mapped to log_2_CPM. Edge width has been mapped to magnitude of correlation.

By combining miRNA-gene interactions from miRTarBase, TarBase, TargetScan, miRWalk and mirDIP followed by selecting only those genes and miRNAs expressed in our TM experimental datasets [16,20], a combined database of miRNA-gene interactions was created. Using this combined interaction database together with the list of 689 differentially expressed genes and 52 differentially expressed miRNAs, a set of possibly interacting miRNA-gene pairs was determined. This set was further purified by adding the constraint of opposite fold-change: if expression of the regulated gene is increased, expression of the regulating miRNA should be decreased and vice versa. Because both mRNA and miRNA species were determined for the same sample, also the correlation of expression of mRNA and miRNA over samples could be determined. This correlation was used as part of an interaction score but not as a filter. The procedure resulted in a bipartite network consisting of 52 miRNAs and 466 genes connected by 1202 likely interacting miRNA-gene pairs, see Fig 2 and S2 Table. Due to the constraint of inverse expression, the network consists of (at least) two unconnected subnetworks, one for up-regulated genes regulated by down-regulated miRs and one for down-regulated genes regulated by up-regulated miRNAs. In addition to this standard interactome which only consists of genes and miRs for which the false discovery rate FDR<0.05 also an enhanced interactome was determined in which the p-value per interaction was determined and correction for multiple testing was performed on the set of interactions rather than the sets of genes or miRs followed by a selection of those interactions for which FDR<0.05. This procedure resulted in 2151 additional interactions between 912 genes and 85 miRNAs. Of these interactions, 1054 originate from experimental database evidence and 1097 from predicting database evidence. The total (standard + enhanced) interactome consists of 3353 interactions.

MiRNAs with the highest number of interactions (hub-miRs) were hsa-let-7e-5p (113 interactions), hsa-miR-335-5p (100 interactions), hsa-miR-15a-5p (96 interactions), hsa-miR-20a-5p (96 interactions), hsa-miR-218-5p (96 interactions), hsa-miR-181a-5p (94 interactions), hsa-miR-29b-3p (90 interactions), hsa-miR-122-5p (87 interactions), hsa-miR-19b-3p (87 interactions), hsa-miR-30b-5p (86 interactions), hsa-miR-181b-5p (84 interactions), hsa-miR-182-5p (81 interactions), hsa-miR-125a-3p (80 interactions), hsa-miR-26a-5p (79 interactions), hsa-miR-29c-3p (77 interactions), hsa-miR-195-5p (74 interactions), hsa-miR-204-5p (74 interactions), hsa-miR-29a-3p (74 interactions), hsa-miR-19a-3p (63 interactions) and hsa-miR-744-5p (63 interactions). The average number of interactions per miRNA was 39.0 and each gene was on average regulated by 2.95 miRNAs.

### miRNA regulation of biological processes altered by TGF-β1 in human TM cells

**Fig 3:**
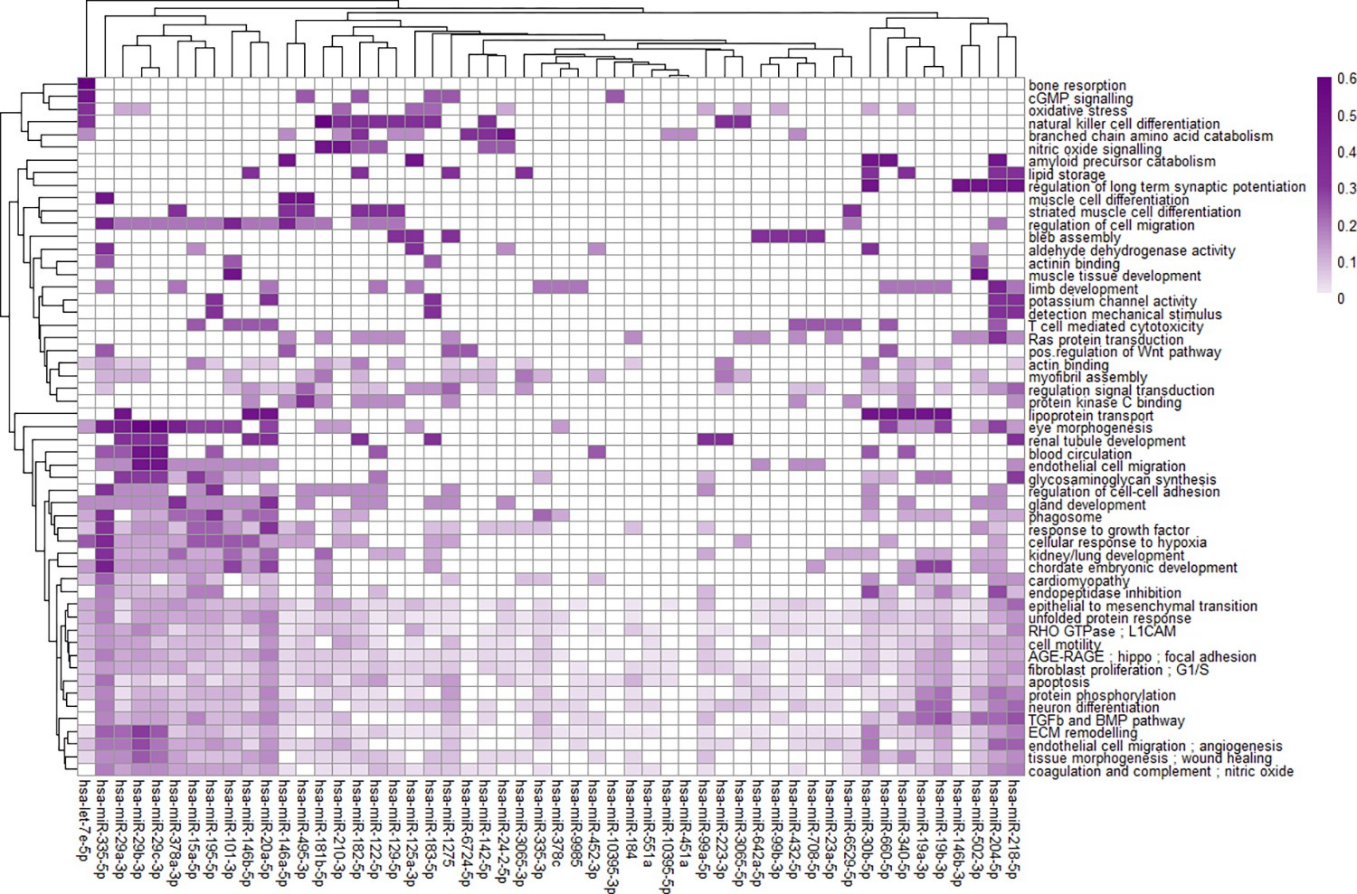
miRNA regulation of biological processes induced by TGF-β1 in human trabecular meshwork cells. The colour intensity of this heatmap corresponds to the number of mRNAs (genes) regulated by a certain miRNA divided by the total number of miRNA-regulated mRNAs for a certain biological process and is therefore a measure of how strongly a certain biological process is regulated by a certain miRNA. Only miRNAs of the standard interactome have been shown (FDR<0.05).

A matrix of miRNAs versus enrichment clusters was created as described in the Methods section, see Fig 3 and S1 Table. It appears that a miRNA cluster consisting of hsa-miR-335-5p, hsa-miR-29a-3p, hsa-miR-29b-3p, hsa-miR-29c-3p, hsa-miR-378a-3p, hsa-miR-15a-5p, hsa-hsa-miR-195-5p, hsa-miR-101-3p, hsa-miR-146b-5p and hsa-miR-20a-5p co-regulate a cluster of functional themes consisting of amongst others “eye morphogenesis”, “regulation of cell migration”, “phagosome” and “cellular response to hypoxia”. Another cluster of functional themes consisting of “epithelial to mesenchymal transition”, “unfolded protein response”, “fibroblast proliferation; G1/S”, “ECM remodelling” and several others was co-regulated by almost all differentially expressed miRNAs except hsa-let-7e-5p and hsa-miR-451a. Other miRNAs that seem to co-regulate similar processes are hsa-miR-204-5p and hsa-miR-218-5p; hsa-miR-19a-3p and hsa-miR-19b-3p; hsa-miR-432-5p and hsa-miR-708-5p and members of the miR-29 family. In the next sections the specific gene-miRNA interactions per functional theme will be investigated for several clusters believed to be of specific interest to XFG.

### miRNA regulation of TGF-β1 induced expression of exfoliation material genes

**Fig 4:**
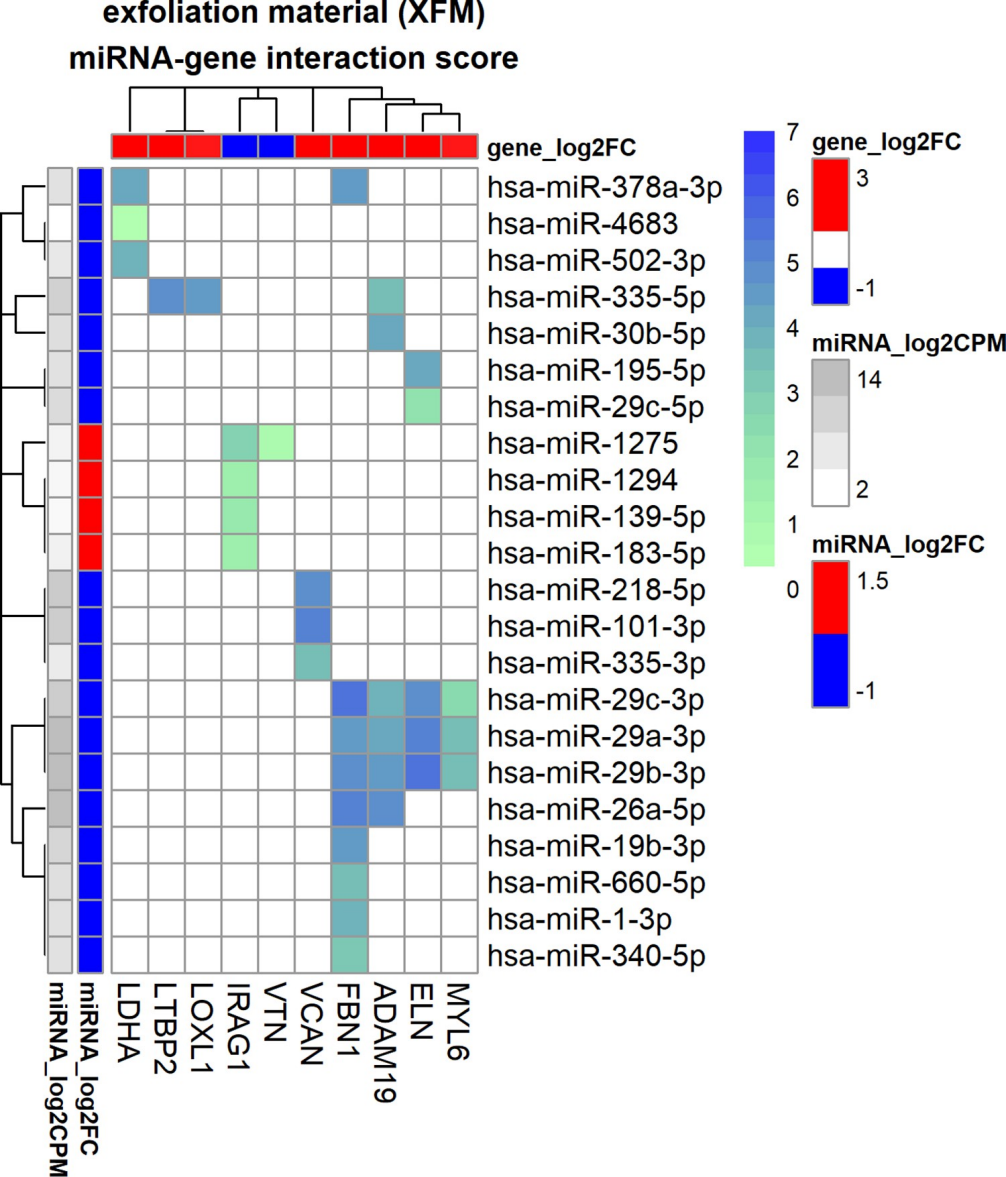
miRNA-gene interactome of exfoliation material genes for human trabecular meshwork cells stimulated by TGF-β1. Heatmap of the interactome in which the heatmap colour maps to the interaction score which is calculated from database evidence, miRNA expression and miRNA-gene correlation using Eq (2). The annotation bars on the left represent miRNA fold-change as log_2_FC (in red/blue) and log_2_CPM (grey-scale) and the annotation bar at the top represents log_2_FC of genes (in red/blue).

Our analysis showed that the expression of 10 exfoliation material (XFM) genes may be regulated by 22 miRNAs via 24 interactions in the standard interactome and 12 additional interactions in the enhanced interactome, see Fig 4 and Table 1. Expression of fibrillin 1 (*FBN1*), *ADAM19*, elastin (*ELN*) and myosin light chain 6 (*MYL6*) was regulated by the miR-29 family (hsa-miR-29a-3p, hsa-miR-29b-3p, hsa-miR-29c-3p). In addition, *ELN* was also regulated by hsa-miR-195-5p and possibly hsa-miR-29c-5p (enhanced interactome) and *ADAM19* was also regulated by hsa-miR-335-5p and hsa-miR-30b-5p. *FBN1* was regulated by the miR-29 family members and also hsa-miR-19b-3p, hsa-miR-660-5p, hsa-miR-1-3p and hsa-miR-340-5p of which hsa-miR-29c-3p had the strongest correlation (-0.93) and highest interaction score (S = 5.5) and substantial interaction evidence and miR molarity. Versican (*VCAN*) was regulated by hsa-miR-101-3p, hsa-miR-335-3p, hsa-miR-218-5p of which hsa-miR-101-3p had the highest interaction score (S = 5.3) due to good correlation combined with strong database evidence and miRNA molarity. Vitronectin (*VTN*) may be regulated by hsa-miR-1275 (enhanced interactome, predicted database interaction), *IRAG1* may be regulated by hsa-miR-1275, hsa-miR-139-5p, hsa-miR-183-5p and hsa-miR-1294 of which hsa-miR-1275 showed the best correlation and all interactions originate from predicting databases. Both lysyl oxidase like 1 (*LOXL1*) and Latent Transforming Growth Factor Beta Binding Protein 2 (*LTBP2*) may be regulated by hsa-miR-335-5p. *LDHA* was regulated by hsa-miR-4683, hsa-miR-378a-3p and hsa-miR-502-3p of which hsa-miR-378a-3p showed the highest interaction score (S = 4.0).

**Table 1:** Interactome of exfoliation material genes for human trabecular meshwork cells stimulated by TGF-β1.

Gene	Interactions
*ADAM19*	hsa-miR-29a-3p, hsa-miR-29b-3p, hsa-miR-29c-3p, hsa-miR-335-5p, hsa-miR-30b-5p, (hsa-miR-26a-5p)
*ELN*	hsa-miR-195-5p, hsa-miR-29a-3p, hsa-miR-29b-3p, hsa-miR-29c-3p, (hsa-miR-29c-5p)
*FBN1*	hsa-miR-340-5p, hsa-miR-660-5p, hsa-miR-19b-3p, hsa-miR-29a-3p, hsa-miR-29b-3p, hsa-miR-29c-3p, hsa-miR-378a-3p, (hsa-miR-1-3p, hsa-miR-26a-5p)
*IRAG1*	hsa-miR-1275, hsa-miR-183-5p, (hsa-miR-139-5p, hsa-miR-1294)
*LDHA*	hsa-miR-378a-3p, hsa-miR-502-3p, (hsa-miR-4683)
*LOXL1*	(hsa-miR-335-5p)
*LTBP2*	hsa-miR-335-5p
*MYL6*	(hsa-miR-29a-3p, hsa-miR-29b-3p, hsa-miR-29c-3p)
*VCAN*	hsa-miR-101-3p, hsa-miR-335-3p, hsa-miR-218-5p
*VTN*	(hsa-miR-1275)

Interactions are presented as standard interactions, for which false discovery rate FDR<0.05 for both genes and miRs, and enhanced interactions between brackets for which FDR<0.05 of the interaction.

### miRNA regulation of TGF-β1 induced extracellular matrix remodelling

**Fig 5:**
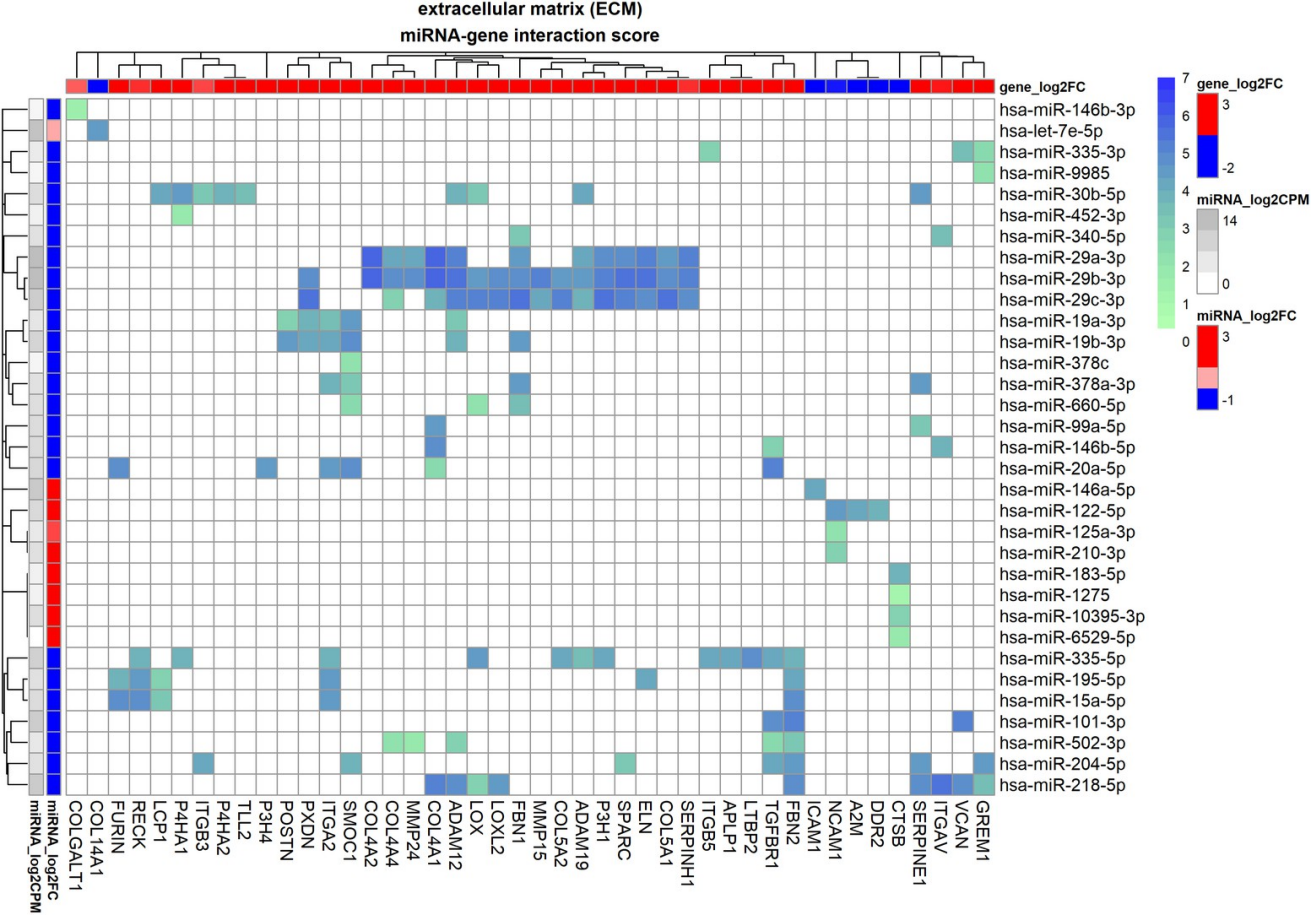
Standard miRNA-gene interactome of extracellular matrix genes for human trabecular meshwork cells stimulated by TGF-β1. Heatmap of the interactome in which the heatmap colour maps to interaction score which is calculated from database evidence, miRNA expression and miRNA-gene correlation using Eq (2). The annotation bars on the left represent miRNA fold-change as log_2_FC (in red/blue) and log_2_CPM (grey-scale) and the annotation bar at the top represents log_2_FC of genes (in red/blue).

This relatively large sub-interactome contains 250 interactions between 60 miRNAs and 72 genes all involved in extracellular matrix remodelling. The standard interactome showed 149 interactions between 44 genes and 33 miRs while the enhanced interactome showed 101 interactions between 50 genes and 52 miRs, see Fig 5 and Table 2. As shown in Fig 5 a large cluster consists of miR-29 family members miR-29a-3p, miR-29b-3p and miR-29c-3p which contribute to the regulation of several collagen species *COL4A1*, *COL4A2*, *COL4A4*, *COL5A1*, *COL5A2* (and additional species such as *COL1A1* regulated by hsa-miR-218-5p, hsa-miR-29b-3p), metallopeptidases *ADAM12*, *ADAM19*, *MMP15* and *MMP24*, and amine oxidases *LOX* and *LOXL2*. In addition, *SPARC*, *P3H1*, fibrillin 1 (*FBN1*), elastin (*ELN*) and *SERPINH1* were found to be members of this cluster.

**Table 2:** Standard miRNA-gene interactome of extracellular matrix genes for human trabecular meshwork cells stimulated by TGF-β1.

Gene	Interactions
*A2M*	hsa-miR-122-5p
*ADAM12*	hsa-miR-19a-3p, hsa-miR-19b-3p, hsa-miR-30b-5p, hsa-miR-218-5p, hsa-miR-29a-3p, hsa-miR-29b-3p, hsa-miR-29c-3p, hsa-miR-502-3p
*ADAM19*	hsa-miR-29a-3p, hsa-miR-29b-3p, hsa-miR-29c-3p, hsa-miR-335-5p, hsa-miR-30b-5p
*APLP1*	hsa-miR-335-5p
*COL14A1*	hsa-let-7e-5p
*COL4A1*	hsa-miR-20a-5p, hsa-miR-99a-5p, hsa-miR-146b-5p, hsa-miR-218-5p, hsa-miR-29a-3p, hsa-miR-29b-3p, hsa-miR-29c-3p
*COL4A2*	hsa-miR-29a-3p, hsa-miR-29b-3p
*COL4A4*	hsa-miR-29a-3p, hsa-miR-29b-3p, hsa-miR-29c-3p, hsa-miR-502-3p
*COL5A1*	hsa-miR-29a-3p, hsa-miR-29b-3p, hsa-miR-29c-3p
*COL5A2*	hsa-miR-335-5p, hsa-miR-29b-3p, hsa-miR-29c-3p
*COLGALT1*	hsa-miR-146b-3p
*CTSB*	hsa-miR-10395-3p, hsa-miR-6529-5p, hsa-miR-1275, hsa-miR-183-5p
*DDR2*	hsa-miR-122-5p
*ELN*	hsa-miR-195-5p, hsa-miR-29a-3p, hsa-miR-29b-3p, hsa-miR-29c-3p
*FBN1*	hsa-miR-340-5p, hsa-miR-660-5p, hsa-miR-19b-3p, hsa-miR-29a-3p, hsa-miR-29b-3p, hsa-miR-29c-3p, hsa-miR-378a-3p
*FBN2*	hsa-miR-335-5p, hsa-miR-101-3p, hsa-miR-15a-5p, hsa-miR-195-5p, hsa-miR-204-5p, hsa-miR-218-5p, hsa-miR-502-3p
*FURIN*	hsa-miR-15a-5p, hsa-miR-195-5p, hsa-miR-20a-5p
*ICAM1*	hsa-miR-146a-5p
*ITGA2*	hsa-miR-19a-3p, hsa-miR-19b-3p, hsa-miR-20a-5p, hsa-miR-335-5p, hsa-miR-378a-3p, hsa-miR-15a-5p, hsa-miR-195-5p
*ITGAV*	hsa-miR-146b-5p, hsa-miR-340-5p, hsa-miR-218-5p
*ITGB3*	hsa-miR-30b-5p, hsa-miR-204-5p
*ITGB5*	hsa-miR-335-3p, hsa-miR-335-5p
*LCP1*	hsa-miR-15a-5p, hsa-miR-195-5p, hsa-miR-30b-5p
*LOX*	hsa-miR-218-5p, hsa-miR-30b-5p, hsa-miR-335-5p, hsa-miR-660-5p, hsa-miR-29b-3p, hsa-miR-29c-3p
*LOXL2*	hsa-miR-218-5p, hsa-miR-29b-3p, hsa-miR-29c-3p
*MMP15*	hsa-miR-29c-3p, hsa-miR-29b-3p
*MMP24*	hsa-miR-502-3p, hsa-miR-29a-3p, hsa-miR-29b-3p
*NCAM1*	hsa-miR-125a-3p, hsa-miR-122-5p, hsa-miR-210-3p
*P3H1*	hsa-miR-29a-3p, hsa-miR-29b-3p, hsa-miR-29c-3p, hsa-miR-335-5p
*P4HA1*	hsa-miR-335-5p, hsa-miR-452-3p, hsa-miR-30b-5p
*P4HA2*	hsa-miR-30b-5p
*POSTN*	hsa-miR-19a-3p, hsa-miR-19b-3p
*PXDN*	hsa-miR-19a-3p, hsa-miR-19b-3p, hsa-miR-29b-3p, hsa-miR-29c-3p
*RECK*	hsa-miR-335-5p, hsa-miR-15a-5p, hsa-miR-195-5p
*SERPINE1*	hsa-miR-204-5p, hsa-miR-378a-3p, hsa-miR-218-5p, hsa-miR-30b-5p, hsa-miR-99a-5p
*SERPINH1*	hsa-miR-29a-3p, hsa-miR-29b-3p, hsa-miR-29c-3p
*SMOC1*	hsa-miR-378a-3p, hsa-miR-660-5p, hsa-miR-19a-3p, hsa-miR-19b-3p, hsa-miR-204-5p, hsa-miR-20a-5p, hsa-miR-378c
*SPARC*	hsa-miR-204-5p, hsa-miR-29a-3p, hsa-miR-29b-3p, hsa-miR-29c-3p
*TLL2*	hsa-miR-30b-5p
*VCAN*	hsa-miR-101-3p, hsa-miR-335-3p, hsa-miR-218-5p

All interactions are standard interactions, for which false discovery rate FDR<0.05 for both genes and miRs.

Prolyl 4-Hydroxylase Subunit *P4HA1* was shown to be regulated by hsa-miR-335-5p, hsa-miR-452-3p, hsa-miR-30b-5p while *P4HA2* was regulated by hsa-miR-30b-5p. Peroxidasin (*PXDN*), involved in ROS-dependent crosslinking of basement membrane component *COL4*, was shown to be regulated by hsa-miR-19a-3p, hsa-miR-19b-3p, hsa-miR-29b-3p, hsa-miR-29c-3p. *FURIN*, an endoprotease of the TGF-β1 precursor, was shown to be upregulated by downregulation of hsa-miR-15a-5p, hsa-miR-195-5p, hsa-miR-20a-5p. *SMOC1* was indicated to be mainly regulated by hsa-miR-19a-3p, hsa-miR-19b-3p, hsa-miR-204-5p and hsa-miR-20a-5p.

Cell adhesion molecule *NCAM1* was indicated to be regulated by hsa-miR-125a-3p, hsa-miR-122-5p, hsa-miR-210-3p of which hsa-miR-122-5p showed the highest interaction score (S = 4.6) due to good correlation (r = -0.7) and strong interaction database evidence. *ICAM1* was found to be regulated by hsa-miR-146a-5p. In the integrin family, *ITGAV* was found to be regulated by hsa-miR-146b-5p, hsa-miR-340-5p and hsa-miR-218-5p of which the latter has the highest interaction score. *ITGA2* was regulated by hsa-miR-19a-3p, hsa-miR-19b-3p, hsa-miR-20a-5p, hsa-miR-335-5p, hsa-miR-378a-3p, hsa-miR-15a-5p and hsa-miR-195-5p of which hsa-miR-15a-5p and hsa-miR-195-5p show the strongest correlation. *ITGB3* was regulated by hsa-miR-30b-5p and hsa-miR-204-5p while *ITGB5* was shown to be regulated by hsa-miR-335-5p (experimental database evidence) and by hsa-miR-335-3p (predicted database evidence). From the enhanced interactome, *ITGB1* was indicated to be regulated by hsa-miR-20a-5p, hsa-miR-218-5p and hsa-miR-29b-3p and *ITGB8* was regulated by mostly hsa-miR-181b-5p, hsa-miR-181a-5p, hsa-miR-432-5p, hsa-miR-708-5p and hsa-let-7e-5p.

### miRNA regulation of TGF-β1 induced expression of signal molecules and downstream pathways

**Fig 6:**
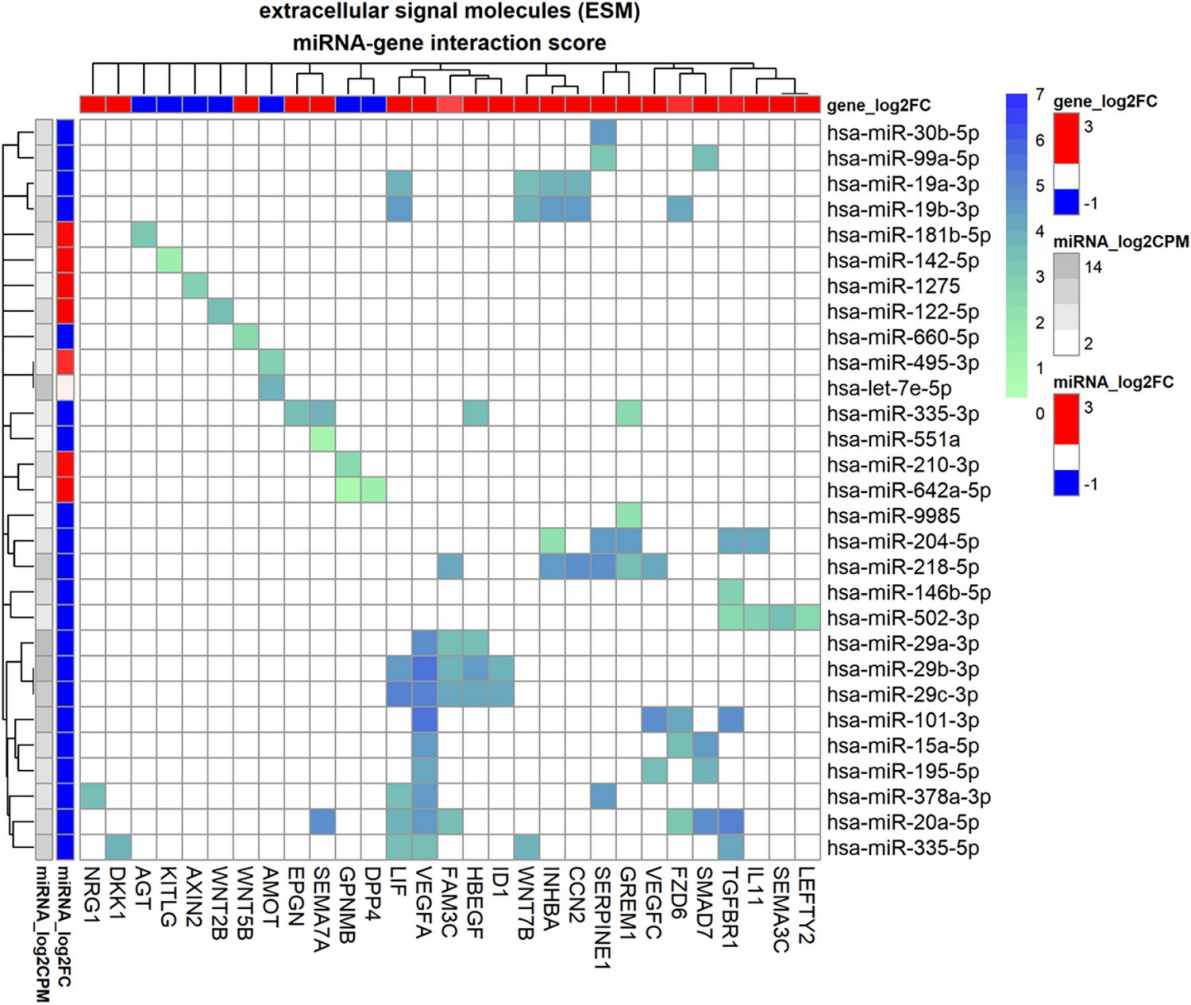
miRNA-gene interactome of genes coding for extracellular signal molecules for human trabecular meshwork cells stimulated by TGF-β1. Heatmap of the interactome in which the heatmap colour maps to interaction score which is calculated from database evidence, miRNA expression and miRNA-gene correlation using Eq (2). The annotation bars on the left represent miRNA fold-change as log2FC (in red/blue) and log2CPM (grey-scale) and the annotation bar at the top represents log2FC of genes (in red/blue).

The interactome analysis resulted in a total of 188 interactions between 63 miRs and 50 genes functionally enriched in signal molecules and their pathways. The total subset of interactions consisted of 83 standard interactions between 29 genes and 29 miRs and 105 additional interactions between 41 genes and 55 miRs from the enhanced interactome, see Fig 6 and Table 3. Fig 6 shows the interactions resulting from the standard interactome method. Starting with the canonical TGF-β pathway, receptor protein gene *TGFBR1* was regulated by a large number of miRNAs namely hsa-miR-101-3p, hsa-miR-204-5p, hsa-miR-20a-5p and hsa-miR-335-5p. When considering the correlation in expression and molarity the key regulators are likely hsa-miR-101-3p, hsa-miR-204-5p and hsa-miR-20a-5p. Gremlin1 (*GREM1*) was regulated by hsa-miR-204-5p, hsa-miR-218-5p and perhaps hsa-miR-335-5p. Of these miRNAs, hsa-miR-204-5p has the highest correlation and is the only interaction that originates from an experimental database. *LEFTY2* was indicated to be regulated by hsa-miR-502-3p (only predicted database evidence). When considering standard interactions, *SMAD7* was regulated by hsa-miR-15a-5p, hsa-miR-195-5p, hsa-miR-20a-5p and hsa-miR-99a-5p. Of these miRNAs, both hsa-miR-15a-5p and hsa-miR-20a-5p had high interaction scores. *SMAD3* appeared in the enhanced interactome (not shown in Fig 6), and a key regulator was hsa-miR-708-5p (S = 4.0). Another miRNA that may regulate *SMAD3* is hsa-miR-744-5p. In the standard interactome, *CCN2* (also called connective tissue growth factor: *CTGF*) was regulated by hsa-miR-19a-3p, hsa-miR-19b-3p and hsa-miR-218-5p. In addition, the enhanced interactome showed regulation by hsa-miR-18a-5p and hsa-miR-26a-5p. Of these miRNAs, hsa-miR-218-5p had the highest interaction score (S = 4.9) due to good correlation (r = -0.6) and high molarity. Moving to genes involved in the Wnt pathway, *WNT2B* was indicated to be regulated by hsa-miR-122-5p (only predicted database evidence). Furthermore, *WNT5B* was regulated by hsa-miR-660-5p and *WNT7B* was regulated by hsa-miR-335-5p, hsa-miR-19a-3p, hsa-miR-19b-3p, hsa-miR-378a-5p and hsa-miR-505-5p which were all experimentally verified interactions. *DKK1* was regulated by hsa-miR-335-5p and hsa-miR-1-3p. Frizzled class receptor 6 (*FZD6*) was regulated by hsa-miR-101-3p, hsa-miR-15a-5p, hsa-miR-19b-3p, hsa-miR-20a-5p and, from the enhanced interactome, also hsa-miR-21-5p. Of these miRNAs the interaction score was strongest for hsa-miR-21-5p (S = 5.6). *AXIN2* was regulated by hsa-miR-1275 with high interaction confidence and strong correlation (r = -0.95) and S = 2.9.

**Table 3:** miRNA-gene interactome of extracellular signal molecule (ESM) genes for human trabecular meshwork cells stimulated by TGF-β1.

Gene	Interactions
*AGT*	hsa-miR-181b-5p
*AMOT*	hsa-miR-495-3p, hsa-let-7e-5p
*AXIN2*	hsa-miR-1275
*CCN2*	hsa-miR-19a-3p, hsa-miR-19b-3p, hsa-miR-218-5p
*DKK1*	hsa-miR-335-5p
*DPP4*	hsa-miR-642a-5p
*EPGN*	hsa-miR-335-3p
*FAM3C*	hsa-miR-20a-5p, hsa-miR-218-5p, hsa-miR-29a-3p, hsa-miR-29b-3p, hsa-miR-29c-3p
*FZD6*	hsa-miR-101-3p, hsa-miR-15a-5p, hsa-miR-19b-3p, hsa-miR-20a-5p
*GPNMB*	hsa-miR-210-3p, hsa-miR-642a-5p
*GREM1*	hsa-miR-218-5p, hsa-miR-9985, hsa-miR-204-5p, hsa-miR-335-3p
*HBEGF*	hsa-miR-29a-3p, hsa-miR-29b-3p, hsa-miR-29c-3p, hsa-miR-335-3p
*ID1*	hsa-miR-29b-3p, hsa-miR-29c-3p
*IL11*	hsa-miR-204-5p, hsa-miR-502-3p
*INHBA*	hsa-miR-204-5p, hsa-miR-19a-3p, hsa-miR-19b-3p, hsa-miR-218-5p
*KITLG*	hsa-miR-142-5p
*LEFTY2*	hsa-miR-502-3p
*LIF*	hsa-miR-20a-5p, hsa-miR-335-5p, hsa-miR-378a-3p, hsa-miR-19a-3p, hsa-miR-19b-3p, hsa-miR-29b-3p, hsa-miR-29c-3p
*NRG1*	hsa-miR-378a-3p
*SEMA3C*	hsa-miR-502-3p
*SEMA7A*	hsa-miR-551a, hsa-miR-20a-5p, hsa-miR-335-3p
*SERPINE1*	hsa-miR-204-5p, hsa-miR-378a-3p, hsa-miR-218-5p, hsa-miR-30b-5p, hsa-miR-99a-5p
*SMAD7*	hsa-miR-15a-5p, hsa-miR-195-5p, hsa-miR-20a-5p, hsa-miR-99a-5p
*TGFBR1*	hsa-miR-146b-5p, hsa-miR-101-3p, hsa-miR-204-5p, hsa-miR-20a-5p, hsa-miR-335-5p, hsa-miR-502-3p
*VEGFA*	hsa-miR-335-5p, hsa-miR-378a-3p, hsa-miR-101-3p, hsa-miR-15a-5p, hsa-miR-195-5p, hsa-miR-20a-5p, hsa-miR-29a-3p, hsa-miR-29b-3p, hsa-miR-29c-3p
*VEGFC*	hsa-miR-101-3p, hsa-miR-195-5p, hsa-miR-218-5p
*WNT2B*	hsa-miR-122-5p
*WNT5B*	hsa-miR-660-5p
*WNT7B*	hsa-miR-335-5p, hsa-miR-19a-3p, hsa-miR-19b-3p

All interactions are standard interactions, for which false discovery rate FDR<0.05 for both genes and miRs.

The expression of interleukin *IL11* was regulated by hsa-miR-204-5p and hsa-miR-502-3p of which hsa-miR-204-5p showed highest interaction score (S = 4.1) due to strong correlation (-0.67) and strong database evidence. Cytokine *LIF* was regulated by hsa-miR-19a-3p, hsa-miR-19b-3p, hsa-miR-20a-5p (best correlation) and miR-29c-3p. Furthermore, the enhanced interactome showed regulation by hsa-miR-18a-5p and hsa-miR-26a-5p so both the 17–92 cluster and the miR-29 family seemed to be involved in regulating *LIF*. Growth factor *EPGN* may be regulated by hsa-miR-335-3p. The enhanced interactome showed regulation of growth factor *FGF2* by hsa-miR-101-3p, hsa-miR-15a-5p, hsa-miR-19b-3p and hsa-miR-218-5p. These interactions all originated from experimental databases. *EGFR* ligand *HBEGF* was regulated by hsa-miR-29a-3p, hsa-miR-29b-3p, hsa-miR-29c-3p and hsa-miR-335-5p and hsa-miR-29c-3p showed the strongest correlation in expression. *VEGFA* was regulated by a large number of miRNAs of which hsa-miR-378-3p and hsa-miR-101-3p showed the highest interaction score (S = 5.5) but hsa-miR-15-5p, hsa-miR-195-5p, hsa-miR-20a-5p and hsa-miR-29c-3p also showed good correlation. *VEGFC* was regulated by hsa-miR-15-5p and hsa-miR-195-5p as well and in addition by hsa-miR-218-5p. Angiotensinogen (*AGT*) was indicated to be regulated by hsa-miR-181b-5p (predicted interaction, not experimental).

### miRNAs contribute to the regulation of oxidative stress in response to TGF-β1 in human TM cells

**Fig 7:**
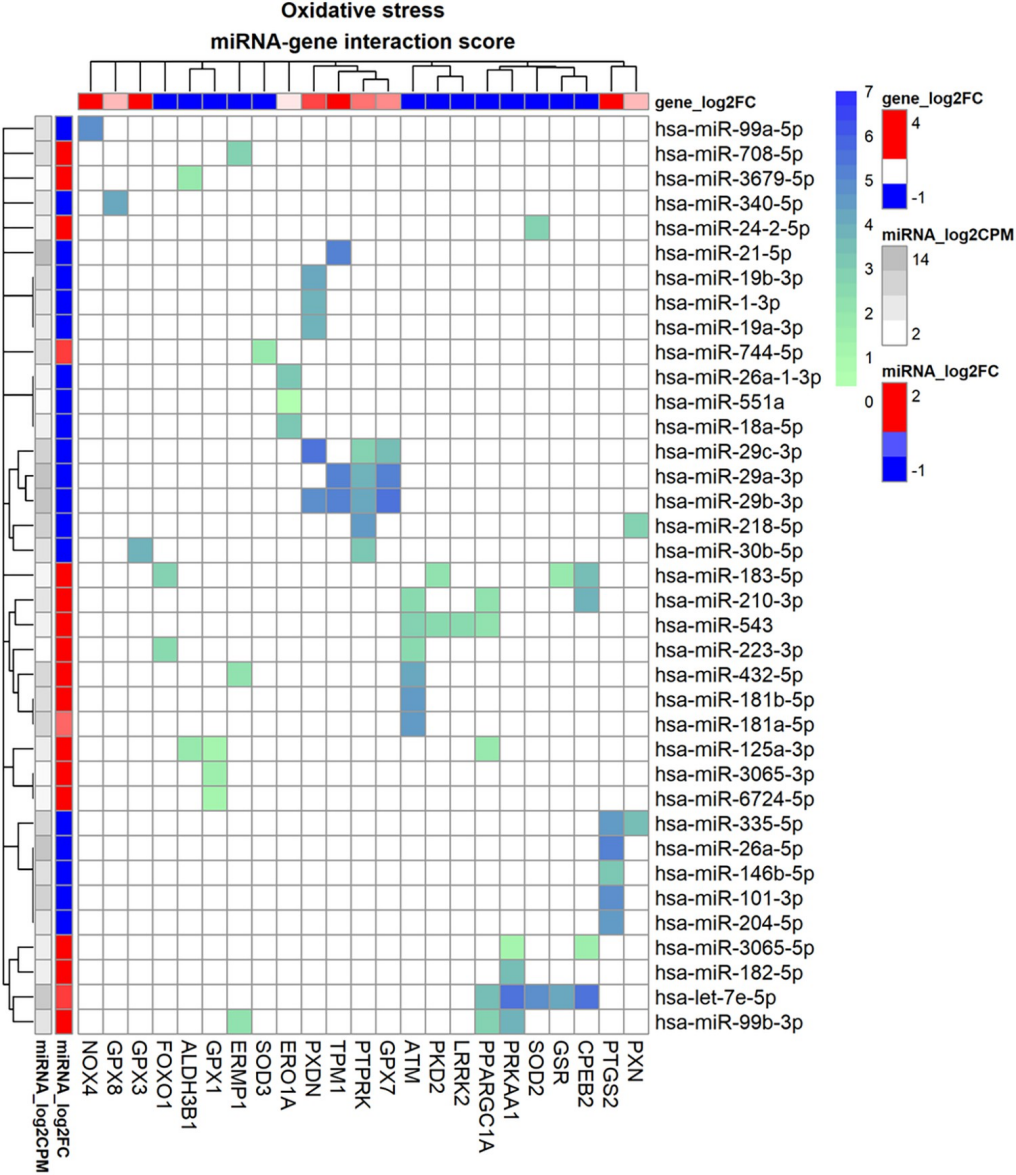
miRNA-gene interactome of genes involved in oxidative stress for human trabecular meshwork cells stimulated by TGF-β1. Heatmap of the interactome in which the heatmap colour maps to interaction score which is calculated from database evidence, miRNA expression and miRNA-gene correlation using Eq (2). The annotation bars on the left represent miRNA fold-change as log_2_FC (in red/blue) and log_2_CPM (grey-scale) and the annotation bar at the top represents log_2_FC of genes (in red/blue).

The interactome analysis indicated that miRNAs contribute to the regulation of mRNA expression of several genes that are associated with oxidative stress, antioxidant activity and oxidative activity. In total 66 interactions between 23 genes and 37 miRs were found of which 25 interactions were between 11 genes and 19 miRs in the standard interactome and 41 interactions between 18 genes and 29 miRs in the enhanced interactome, see Fig 7 and Table 4 below. NADPH Oxidase 4 (*NOX4*) was regulated by hsa-miR-99a-5p and showed a high interaction score (S = 4.9) and good correlation of -0.91 suggesting that this miRNA may be an important regulator. Superoxide dismutase 2 (*SOD2*) was regulated by amongst others hsa-miR-24-2-5p and hsa-let-7e-5p. Both interactions show up in both experimental and predicting miRNA databases. Superoxide 3 (*SOD3*) may be regulated by hsa-miR-744-5p (this interaction was only supported by predicted database evidence). Of the glutathione peroxidases, *GPX1* was downregulated by hsa-miR-125-3p (high-confidence prediction) and hsa-miR-3065. *GPX7* was downregulated by all 3 members of the miR-29 family: hsa-miR-29a-3p, hsa-miR-29b-3p, hsa-miR-29c-3p (interactions both experimentally verified and predicted database interaction). *GPX3* was upregulated due to downregulation of hsa-miR-30b-5p (predicted by miRWalk and Tarbase) while *GPX8* was upregulated due to downregulation of hsa-miR-340-5p with high interaction score (S = 4.3) and strong correlation (r = -0.75). *GSR* was regulated by hsa-let-7e-5p and hsa-miR-183-5p in which hsa-let-7e-5p had the highest score and molarity, but correlation was not very strong which may indicate that there are other non-miRNA regulators that play a role. *PTGS2* (*COX2*) was regulated by hsa-miR-204-5p, hsa-miR-335-5p, hsa-miR-26a-5p, 101-3p and hsa-miR-146b-5p of which hsa-miR-26a-5p showed the strongest interaction score (S = 5.2). In the enhanced interactome, *ERO1A* was regulated by hsa-miR-551a, hsa-miR-18a-5p and hsa-miR-26a-1-3p with both hsa-miR-18a-5p and hsa-miR-26a-1-3p appearing as possible major regulators; judged by their interaction scores.

**Table 4:** Interactome of genes involved in oxidative stress for human trabecular meshwork cells stimulated by TGF-β1.

Gene	Interactions
*ALDH3B1*	hsa-miR-125a-3p, (hsa-miR-3679-5p)
*ATM*	(hsa-miR-181b-5p, hsa-miR-210-3p, hsa-miR-432-5p, hsa-miR-543, hsa-miR-181a-5p, hsa-miR-223-3p)
*CPEB2*	hsa-miR-183-5p, hsa-miR-3065-5p, hsa-let-7e-5p, hsa-miR-210-3p
*ERMP1*	(hsa-miR-99b-3p, hsa-miR-432-5p, hsa-miR-708-5p)
*ERO1A*	(hsa-miR-551a, hsa-miR-18a-5p, hsa-miR-26a-1-3p)
*FOXO1*	(hsa-miR-183-5p, hsa-miR-223-3p)
*GPX1*	(hsa-miR-125a-3p, hsa-miR-3065-3p, hsa-miR-6724-5p)
*GPX3*	hsa-miR-30b-5p
*GPX7*	(hsa-miR-29a-3p, hsa-miR-29b-3p, hsa-miR-29c-3p)
*GPX8*	hsa-miR-340-5p
*GSR*	(hsa-miR-183-5p, hsa-let-7e-5p)
*LRRK2*	(hsa-miR-543)
*NOX4*	hsa-miR-99a-5p
*PKD2*	hsa-miR-183-5p, (hsa-miR-543)
*PPARGC1A*	hsa-let-7e-5p, hsa-miR-125a-3p, hsa-miR-210-3p, hsa-miR-99b-3p, (hsa-miR-543)
*PRKAA1*	(hsa-miR-3065-5p, hsa-let-7e-5p, hsa-miR-182-5p, hsa-miR-99b-3p)
*PTGS2*	hsa-miR-101-3p, hsa-miR-204-5p, hsa-miR-335-5p, hsa-miR-146b-5p, (hsa-miR-26a-5p)
*PTPRK*	(hsa-miR-29a-3p, hsa-miR-29b-3p, hsa-miR-29c-3p, hsa-miR-30b-5p, hsa-miR-218-5p)
*PXDN*	hsa-miR-19a-3p, hsa-miR-19b-3p, hsa-miR-29b-3p, hsa-miR-29c-3p, (hsa-miR-1-3p)
*PXN*	(hsa-miR-218-5p, hsa-miR-335-5p)
*SOD2*	hsa-let-7e-5p, hsa-miR-24-2-5p
*SOD3*	(hsa-miR-744-5p)
*TPM1*	hsa-miR-29a-3p, hsa-miR-29b-3p, (hsa-miR-21-5p)

Interactions are presented as standard interactions, for which false discovery rate FDR<0.05 for both genes and miRs, and enhanced interactions between brackets for which FDR<0.05 of the interaction.

### miRNA regulation of TGF-β1 induced unfolded protein response

The interactome showed 83 interactions between 32 miRNAs and 22 genes involved in the unfolded protein response. The standard interactome analysis resulted in 35 interactions between 10 genes and 16 miRNAs and the enhanced interactome yielded 48 interactions between 17 genes and 27 miRNAs, see S1 Fig and Table 5 below. The enhanced interactome indicated that *HSPA5* (also known as BiP or *GRP78*) was regulated by hsa-miR-29a-3p, hsa-miR-30b-5p, and hsa-miR-335-5p and all these interactions originated from experimental interaction databases. Of these miRNAs, hsa-miR-30b-5p showed the highest interaction score (S = 4.0). *EIF2AK3* (also known as *PERK*) was regulated by hsa-miR-204-5p, hsa-miR-335-5p, hsa-miR-26a-5p of which hsa-miR-204-5p showed the strongest correlation (r = -0.94) and hsa-miR-26a-5p the highest interaction score (S = 5.4). Both these interactions originated from experimental interaction databases. *HSP90B1*, also known as *GRP94*, was indicated to be controlled by hsa-miR-15a-5p, hsa-miR-218-5p, hsa-miR-101-3p and hsa-miR-335-5p. Heat shock protein *DNAJB11* was regulated by three members of the miR-29 family: hsa-miR-29a-3p, hsa-miR-29b-3p and hsa-miR-29c-3p. UPR-related transcription factor *ATF3* was indicated to be regulated by hsa-miR-181a-2-3p (only predicted evidence). *FKBP14* is involved in collagen protein folding and was indicated to be regulated by hsa-miR-101-3p, hsa-miR-335-5p, hsa-miR-660-5p, hsa-miR-146b-5p, hsa-miR-20a-5p, hsa-miR-29b-3p and hsa-miR-29c-3p. *HYOU1* was indicated to be regulated by hsa-miR-15a-5p (r = -0.88) and hsa-miR-146b-5p (r = -0.43). *HERPUD1* was regulated by hsa-miR-335-5p and hsa-miR-340-5p. Within the cluster of UPR-genes, hsa-miR-335-5p seemed to be a major ‘hub’ miRNA and controlled 10 UPR genes out of 22 including key UPR genes *HSPA5* (BiP) and *EIF2AK3* (*PERK*). This also followed from the miRNA-enrichment matrix analysis described earlier.

**Table 5:** miRNA-gene interactome of genes involved in the Unfolded Protein Response (UPR) for human trabecular meshwork cells stimulated by TGF-β1.

Gene	Interactions
*ATF3*	(hsa-miR-142-3p, hsa-miR-181a-2-3p, hsa-miR-3679-5p)
*CREB3*	(hsa-miR-378a-5p)
*CREB3L2*	(hsa-miR-146b-5p, hsa-miR-19a-3p, hsa-miR-19b-3p, hsa-miR-204-5p, hsa-miR-378a-3p, hsa-miR-29a-3p)
*DNAJB11*	hsa-miR-29a-3p, hsa-miR-29b-3p, hsa-miR-29c-3p
*DNAJB9*	hsa-miR-20a-5p, hsa-miR-335-5p
*DNAJC3*	(hsa-miR-335-5p, hsa-miR-502-5p, hsa-miR-218-5p)
*EIF2AK3*	(hsa-miR-204-5p, hsa-miR-335-5p, hsa-miR-26a-5p)
*EIF2S3*	(hsa-let-7e-5p, hsa-miR-543)
*FKBP14*	hsa-miR-101-3p, hsa-miR-335-5p, hsa-miR-660-5p, hsa-miR-146b-5p, hsa-miR-20a-5p, hsa-miR-29b-3p, hsa-miR-29c-3p, (hsa-miR-29c-5p)
*GFPT1*	(hsa-miR-335-5p, hsa-miR-101-3p, hsa-miR-195-5p, hsa-miR-19a-3p, hsa-miR-19b-3p, hsa-miR-218-5p, hsa-miR-378a-3p)
*HERPUD1*	hsa-miR-335-5p, hsa-miR-340-5p
*HSP90B1*	(hsa-miR-101-3p, hsa-miR-15a-5p, hsa-miR-335-5p, hsa-miR-218-5p)
*HSPA5*	(hsa-miR-335-5p, hsa-miR-29a-3p, hsa-miR-30b-5p)
*HYOU1*	hsa-miR-15a-5p, hsa-miR-335-5p, hsa-miR-146b-5p, hsa-miR-195-5p
*LMNA*	hsa-let-7e-5p, (hsa-miR-744-5p)
*MYDGF*	(hsa-miR-452-3p, hsa-miR-18a-5p)
*PDIA6*	(hsa-miR-15a-5p, hsa-miR-195-5p, hsa-miR-218-5p)
*SERP1*	hsa-miR-204-5p, hsa-miR-20a-5p, hsa-miR-218-5p, (hsa-miR-21-5p, hsa-miR-1-3p, hsa-miR-26a-5p)
*SRPRB*	hsa-miR-146b-5p, hsa-miR-15a-5p, hsa-miR-195-5p, hsa-miR-378a-3p
*SSR1*	hsa-miR-146b-5p, hsa-miR-15a-5p, hsa-miR-195-5p, hsa-miR-19a-3p, hsa-miR-20a-5p, hsa-miR-218-5p, hsa-miR-378a-3p, hsa-miR-660-5p, (hsa-miR-1-3p, hsa-miR-26a-5p)
*SYVN1*	(hsa-miR-1179, hsa-miR-335-5p, hsa-miR-452-3p)
*TPP1*	hsa-let-7e-5p, (hsa-miR-744-5p)

Interactions are presented as standard interactions, for which false discovery rate FDR<0.05 for both genes and miRs, and enhanced interactions between brackets for which FDR<0.05 of the interaction.

### miRNA regulation of TGF-β1 induced changes in transcription factor expression

This subset contains 175 interactions between 53 transcription factors (and some other DNA-binding proteins) and 52 miRs, namely 63 interactions between 18 genes and 30 miRs in the standard interactome and 112 interactions between 46 genes 43 miRs in the enhanced interactome, see S2 Fig and Table 6 below. MiRNAs with the highest numbers of interactions were hsa-miR-129-5p (11), hsa-let-7e-5p (10), hsa-miR-122-5p (9), hsa-miR-495-3p (9), hsa-miR-181a-5p (8), hsa-miR-182-5p (8), hsa-miR-125a-3p (6). It is interesting to see that miRNA hsa-miR-122-5p is highly ranked in this subset because this was the miRNA with the lowest FDR and high fold change (FDR = 9e-15, log2FC = 3.9) in our miRNA dataset. Forkhead box D1 (*FOXD1*) was indicated to be regulated by hsa-miR-99a-5p, hsa-miR-30b-5p, hsa-miR-378a-3p of which the latter showed the highest interaction score. The enhanced interactome showed that *FOXO1* may be downregulated due to upregulation of hsa-miR-183-5p and hsa-miR-223-3p. Downregulated histone deacetylase 5 *HDAC5*, a co-repressor of *RARA*, was indicated to be regulated by hsa-miR-146a-5p and hsa-miR-495-3p (predicted database evidence only). Epithelial-mesenchymal transition (EMT) associated transcription factor *ZEB1* was indicated to be regulated by hsa-miR-3065-5p, hsa-miR-183-5p, hsa-miR-223-3p in the standard interactome of which hsa-miR-183-5p showed the strongest correlation. *ZEB1* was also indicated to be controlled by hsa-miR-200c-3p but the FDR of this interaction was just above the limit of FDR = 0.05 (0.054) and was therefore pruned. *ZEB1* paralog *ZEB2* was regulated by a different set of miRNAs: hsa-miR-122-5p, hsa-miR-125a-3p, hsa-miR-129-5p, hsa-miR-181a-5p, hsa-miR-182-5p (all in the enhanced interactome).

**Table 6:** miRNA-gene interactome of transcription factors for human trabecular meshwork cells stimulated by TGF-β1.

Gene	Interactions
*AHR*	(hsa-miR-181a-5p, hsa-let-7e-5p)
*ARNT2*	hsa-miR-708-5p, hsa-let-7e-5p, hsa-miR-181b-5p, (hsa-miR-181a-2-3p, hsa-miR-543, hsa-miR-181a-5p)
*ARNTL2*	(hsa-miR-6529-5p, hsa-miR-99b-3p, hsa-miR-744-5p)
*ATF3*	(hsa-miR-142-3p, hsa-miR-181a-2-3p, hsa-miR-3679-5p)
*ATOH8*	hsa-miR-20a-5p
*BACH1*	(hsa-miR-543, hsa-let-7e-5p)
*BCL6*	(hsa-miR-1275)
*BHLHE40*	(hsa-miR-195-5p, hsa-miR-15a-5p)
*BHLHE41*	(hsa-miR-122-5p, hsa-miR-1275, hsa-miR-146a-5p, hsa-miR-182-5p)
*CEBPD*	(hsa-let-7e-5p)
*CREB3*	(hsa-miR-378a-5p)
*CREB3L2*	(hsa-miR-146b-5p, hsa-miR-19a-3p, hsa-miR-19b-3p, hsa-miR-204-5p, hsa-miR-378a-3p, hsa-miR-29a-3p)
*CREB5*	(hsa-miR-122-5p, hsa-miR-129-5p, hsa-miR-182-5p)
*E2F7*	hsa-miR-340-5p, hsa-miR-15a-5p, hsa-miR-195-5p, hsa-miR-20a-5p, hsa-miR-29a-3p, hsa-miR-29b-3p, hsa-miR-29c-3p, hsa-miR-30b-5p, hsa-miR-378a-3p, (hsa-miR-26a-5p)
*EMX2*	(hsa-miR-1307-3p, hsa-miR-432-5p)
*ETV1*	(hsa-miR-122-5p, hsa-miR-142-5p, hsa-miR-210-3p, hsa-miR-642a-5p, hsa-miR-129-5p)
*FOSL2*	(hsa-miR-125a-3p, hsa-miR-181b-5p, hsa-miR-495-3p)
*FOXD1*	hsa-miR-99a-5p, hsa-miR-30b-5p, hsa-miR-378a-3p
*FOXO1*	(hsa-miR-183-5p, hsa-miR-223-3p)
*GLIS3*	(hsa-miR-129-5p, hsa-miR-495-3p, hsa-miR-642a-5p)
*HDAC5*	hsa-miR-146a-5p, hsa-miR-495-3p
*HHEX*	(hsa-miR-223-3p)
*HMGA2*	(hsa-miR-122-5p, hsa-miR-129-5p)
*IKZF2*	hsa-let-7e-5p, hsa-miR-122-5p, hsa-miR-129-5p, hsa-miR-146a-5p, hsa-miR-210-3p, hsa-miR-432-5p, (hsa-miR-574-3p)
*IRF2*	(hsa-miR-495-3p, hsa-miR-182-5p)
*KCNIP3*	(hsa-miR-432-5p, hsa-miR-129-5p)
*KLF10*	hsa-miR-378a-3p, hsa-miR-19a-3p, hsa-miR-19b-3p, hsa-miR-20a-5p, hsa-miR-218-5p, hsa-miR-30b-5p, hsa-miR-340-5p, (hsa-miR-26a-5p)
*KLF13*	(hsa-miR-122-5p, hsa-miR-129-5p, hsa-miR-146a-5p, hsa-miR-182-5p, hsa-miR-574-3p)
*KLF15*	hsa-miR-181b-5p, hsa-miR-182-5p, (hsa-miR-181a-5p)
*LITAF*	hsa-miR-184, hsa-miR-182-5p, hsa-miR-23a-5p
*MAFK*	hsa-miR-101-3p, hsa-miR-15a-5p
*MYPOP*	(hsa-miR-26a-5p)
*NACC2*	(hsa-miR-181b-5p)
*NFIL3*	hsa-miR-183-5p, hsa-miR-3065-5p
*NFIX*	(hsa-miR-210-3p, hsa-miR-223-3p, hsa-miR-23a-5p, hsa-miR-1275, hsa-miR-24-2-5p, hsa-miR-744-5p)
*NR2F2*	(hsa-miR-432-5p, hsa-miR-495-3p)
*PATZ1*	hsa-miR-122-5p, hsa-miR-708-5p, (hsa-miR-142-3p)
*PBX1*	hsa-miR-210-3p, hsa-let-7e-5p, hsa-miR-125a-3p, hsa-miR-181b-5p, (hsa-miR-181a-5p, hsa-miR-543)
*PITX1*	(hsa-miR-129-5p)
*PRDM5*	(hsa-let-7e-5p, hsa-miR-125a-3p)
*PRRX1*	hsa-miR-125a-3p, hsa-miR-182-5p, hsa-let-7e-5p
*RORB*	(hsa-miR-210-3p, hsa-miR-432-5p, hsa-miR-495-3p, hsa-miR-181a-5p, hsa-miR-181b-5p)
*SKIL*	hsa-miR-218-5p, hsa-miR-15a-5p, hsa-miR-19a-3p, hsa-miR-19b-3p, hsa-miR-20a-5p, hsa-miR-30b-5p, (hsa-miR-532-5p)
*SMAD3*	(hsa-miR-125a-3p, hsa-miR-139-3p, hsa-miR-142-5p, hsa-miR-708-5p, hsa-miR-744-5p)
*SREBF1*	(hsa-let-7e-5p, hsa-miR-495-3p, hsa-miR-744-5p)
*STAT5A*	hsa-miR-223-3p, (hsa-miR-1307-3p)
*STAT5B*	(hsa-miR-99b-5p)
*TFAP4*	(hsa-miR-495-3p, hsa-miR-3065-5p)
*TWIST1*	(hsa-miR-210-3p, hsa-miR-3065-5p, hsa-miR-3679-5p, hsa-miR-129-5p, hsa-miR-181a-5p, hsa-miR-543)
*ZEB1*	hsa-miR-3065-5p, hsa-miR-183-5p, hsa-miR-223-3p, (hsa-miR-142-3p, hsa-miR-139-5p)
*ZEB2*	(hsa-miR-122-5p, hsa-miR-125a-3p, hsa-miR-129-5p, hsa-miR-181a-5p, hsa-miR-182-5p)
*ZFHX4*	hsa-let-7e-5p, hsa-miR-129-5p, hsa-miR-495-3p, hsa-miR-183-5p, (hsa-miR-181a-5p, hsa-miR-543)
*ZNF395*	(hsa-miR-122-5p)

Interactions are presented as standard interactions, for which false discovery rate FDR<0.05 for both genes and miRs, and enhanced interactions between brackets for which FDR<0.05 of the interaction.

### miRNA–transcription factor loops

Using the method described earlier, two double negative miRNA—| TF—| miRNA loops were identified: hsa-miR-15a-5p forms a double negative feedback loop with *E2F7* and hsa-miR-183-5p forms a double-negative feedback loop with *ZEB1*. See S3 Table for TF—| miRNA interactions and S2 Fig for the corresponding miRNA—| TF interactions.

## Discussion

Our analysis of miRNA-mRNA interactions provides a bioinformatics-based hypothesis-generating prediction of part of the regulatory mechanisms causing differential gene expression in normal human TM tissue when treated with TGF-β1. Normal human TM tissue treated with TGF-β1 shows overlap with features of XFG pathogenesis and may therefore serve as a simplified model for the TM in this condition [16]. As such the miRNA-gene interactome in this work may also partially model the regulation of changes in gene expression observed in XFG and may be a first step in explaining the regulatory function of differentially expressed miRNA species in XFG when compared to normal TM tissue. Many studies have been performed to determine what miRNA species are differentially expressed in XFG and other forms of glaucoma while other studies focussed on the regulatory targets for one species of miRNA [2,19]. To our knowledge, this is the first study that combines genome-wide miRNA and mRNA (gene) expression into one genome-wide interactome for normal TM cells treated with TGF-β1.

It is interesting to compare those miRNAs with the highest number of miRNA-gene interactions (hub-miRs) found in this study with miRNAs previously associated with XFG or glaucoma. MiRNA hsa-miR-20a-5p was previously reported to be downregulated in senescent TM cells [38]. hsa-miR-181a-5p was reported to be upregulated in TM cells subjected to mechanical stretch [39]. Changes in hsa-miR-15a-5p were observed in senescent TM cells [38,39]. hsa-miR-19b-3p was not previously associated with glaucoma to our knowledge. hsa-miR-30b-5p has not been reported in the context of glaucoma before but some close relatives have such as hsa-miR-30c-5p in plasma of XFG patients [40]. Upregulation of hsa-miR-122-5p was reported in the aqueous humour [41] and plasma [40] of XFG patients. MiRNA hsa-miR-181b-5p was not previously associated with glaucoma but the other strand hsa-miR-181b-3p has been reported in AH of glaucoma patients [42]. hsa-miR-26a-5p was previously observed in plasma of XFG patients [40]. hsa-miR-218-5p has not been associated with glaucoma before but has been reported in other fibrotic conditions [43].

Downregulation of mir-29 family members hsa-miR-29a-3p, hsa-miR-29b-3p, hsa-miR-29c-3p was previously observed in iridial and ciliary body tissue specimens obtained from XFG patients when compared to controls [44] and in lamina cibrosa (LC) tissue from POAG patients [45]. MiRNA hsa-miR-204-5p was reported to be downregulated in TM tissue subjected to oxidative stress and senescence [18] and in the glaucomatous retina [46] but upregulated in plasma of XFG patients [40]. MiRNA hsa-miR-18a-5p was reported to be downregulated due to stress-induced senescence [38] and altered in plasma of XFG patients [40]. MiRNA hsa-miR-195-5p and hsa-miR-139-5p were reported to be altered due to stress-induced senescence in TM tissue [38].

Previous findings indicate that glaucoma induces differential expression of a large number of miRNAs when compared to normal controls [19] and our results show that a large fraction of those differentially expressed miRNAs also appear in normal TM tissue when treated with TGF-β1. To investigate what the regulatory effect of these differentially expressed miRNA species on gene expression may be, we constructed an interactome describing putative regulatory relationships between differentially expressed miRNAs and oppositely differentially expressed genes.

Coupling functional enrichment of differentially expressed genes to their putative regulating miRNAs indicates that miRNAs appear to be involved in the regulation of all altered biological processes in TM tissue when treated with TGF-β1. Individual miRNA species usually regulate a wide range of genes and biological processes, and each individual biological process or pathway is regulated by several miRNAs. Therefore, it does not usually seem to be possible to pinpoint one key miRNA which is mostly responsible for a change in a biological process. Rather, it seems that groups of genes (modules) or biological processes are co-regulated by the combined effect of multiple co-regulating miRNAs [47]. For example, one previously studied case of co-regulation by multiple miRNAs is the miR-15/107 group [48], which indeed also showed co-regulation of multiple biological processes by members hsa-miR-15a-5p and hsa-miR-195-5p in our results. Another example is the regulation of ECM remodelling by members of the miR-29 family and ECM remodelling [49].

Our results showed upregulation of several XFM components due to downregulation of miR-29 members hsa-miR-29a-3p, hsa-miR-29b-3p and hsa-miR-29c-3p. The miR-29 family plays a key role in regulation of extracellular matrix components [49] and XFM also contains several ECM-related proteins. Regulation of *LOXL1* by hsa-miR-335-5p has not been reported in glaucoma so far but has previously been reported in breast cancer tissue using microarrays so this may be a downstream regulatory effect [50]. Regulation of *ADAM19* by hsa-miR-335-5p as a result of TGF-β1 treatment has been reported in HK-2 cells [51]. Regulation of elastin (*ELN*) by hsa-miR-195-5p has not been reported before in the context of XFG but was experimentally demonstrated in aortic aneurysmal disease [52]. Regulation of versican (*VCAN*) by hsa-miR-218-5p, hsa-miR-101-3p was identified in our results but has not been reported in XFG or other forms of glaucoma to our knowledge. Overall, the miR-29 family and hsa-miR-335-5p have the highest number of interactions in the XFM functional cluster of genes.

Oxidative stress is associated with XFG [11]. Our results show that in the trabecular meshwork, several key genes involved in oxidative stress and antioxidant activity may be regulated by miRNAs in response to TGF-β1 treatment. In agreement with our findings, regulation of *NOX4* by hsa-miR-99a-5p was also reported in human endothelial cells [53]. Also, several glutathione peroxidases are regulated by miRNAs. A bioinformatics study on this topic was performed by [54] which suggested regulation of *GPX3* by hsa-miR-30b-5p and *GPX1* by hsa-miR-125a-3p which agrees with our results. *GPX7* has been reported to be regulated by hsa-miR-29b [55] which is in agreement with our findings. Regulation of peroxidasin *PXDN* by hsa-miR-29a has been experimentally confirmed in T-cells [56]. *PTGS2* (also known as *COX2*) was experimentally shown to be regulated by hsa-miR-26a-5p [57].

Treatment of TM cells with TGF-β1 causes differential expression of several extracellular signal molecules and their related downstream genes [16,58]. Our interactome analysis indicated that TGF-β1 induced differentially expressed miRNAs contribute to this differential gene expression. *TGFBR1* (also known as *ALK5*) regulation by hsa-miR-101-3p was demonstrated previously [59]. *TGFBR1* regulation by hsa-miR-204-5p was also reported in [60] and regulation by hsa-miR-20a-5p was demonstrated previously as well [61] in endothelial cells in agreement with our results. *SMAD3* has been shown to be downregulated by upregulation of hsa-miR-708-5p [62] which agrees with our findings. *SMAD7* was demonstrated to be directly regulated by hsa-miR-15b-5p [63] which supports regulation of *SMAD7* by hsa-miR-15a-5p in our results as these miRNAs share the same seed sequence. *SMAD7* expression is known to be upregulated in response to TGF-β pathway activation via *SMAD3* [64] so it appears that *SMAD7* expression is regulated by both transcription factors and miRNAs in response to TGF-β1 treatment which may point to the existence of a feed-forward motif. *CCN2* (also known as connective tissue growth factor *CTGF*) regulation by hsa-miR-218-5p was demonstrated experimentally [65] which agrees with our results. *CCN2* was also shown to be controlled by hsa-miR-19 members [66] and hsa-miR-18a-5p [21] which also agrees with our results. Vascular Endothelial Growth Factor A *VEGFA* was reported to be regulated by many miRNAs including hsa-miR-101-3p [67] and by members of the miR-29 family [68] which is in keeping with our findings. The Wnt/β-catenin pathway is associated with glaucoma, IOP regulation and EMT [69–71]. Several key genes of the Wnt/β-catenin pathway were reported to be regulated by miRNAs and several key regulators also appear in our results such as hsa-miR-335-5p [72] and hsa-miR-122-5p [73]. Interleukin *IL11* is essential for development of kidney and heart fibrosis and is involved in EMT [74,75]. Regulation of *IL11* by hsa-miR-204-5p was demonstrated previously in TM tissue [76] and other tissue types [77] and also appears in our interactome.

Activation of the unfolded protein response has been implicated in glaucoma [78]. Several key UPR genes have been reported to be regulated by miRNAs [79].

Our results indicate that *EIF2AK3* (also known as *PERK*) is regulated by several miRNAs including hsa-miR-204-5p which agrees with the literature [80]. Regulation of *HSPA5* (also known as BiP, *GRP78*) by miR-30a was reported before [81] and may in part support our finding of regulation of *HSPA5* by hsa-miR-30b-5p. According to our interactome a key miRNA involved in UPR regulation appears to be hsa-miR-335-5p but this has not been explicitly mentioned in literature as yet.

Remodelling of the extracellular matrix is associated with both TM [82] and LC [83] in glaucoma. Regulation of components, enzymes and other proteins of the ECM by miRNAs has been reported extensively in literature [19,23]. Upregulation of collagen species due to downregulation of miRNA-29 family members hsa-miR-29a-3p, hsa-miR-29b-3p and hsa-miR-29c-3p has been reported in several studies [49] and agrees with our findings. In addition, hsa-miR-146b-5p and hsa-miR-218-5p may play a role in regulating these genes. In keeping with our findings, regulation of *SPARC* by miR-29 family members was reported before *in vitro* in TM cells in response to TGF-β2 treatment [84].

Transcription factor *ZEB1* is associated with fibrosis [85–87] and, in agreement with our interactome, has been reported to be regulated by hsa-miR-183-5p and hsa-miR-223-3p with strong experimental evidence [35]. Related transcription factor *ZEB2* is also associated with fibrosis and fibroblast to myofibroblast transdifferentiation [88]. There is experimental evidence that *ZEB2* may be regulated by hsa-miR-181a-5p [89] and furthermore several predictive databases propose regulation by hsa-miR-122-5p but without experimental evidence to date. Kruppel Like Factor 13 *KLF13* is an antifibrotic transcription factor and is downregulated by TGF-β1 treatment in our study [90] and the interactome only showed predicted interactions of *KLF13*. *KLF15* is also antifibrotic and downregulated and inhibits expression of pro-fibrotic cytokine *CCN2* (*CTGF*) [91]. We demonstrated that *KLF15* was downregulated due to TGF-β1 and has previously been reported to be regulated by hsa-miR-181a-5p in six different publications according to Tarbase [34]. Transcription factor *FOXO1* is involved in several stress responses [92] and is regulated by hsa-miR-183-5p [93] and hsa-miR-223-3p [94] with strong experimental evidence which is in agreement with our interactome. Transcription factor *E2F7* is involved in cell cycle control. There is experimental PAR-CLIP based evidence from at least six different sources that *E2F7* can be regulated by hsa-miR-15a-5p, see interaction MIRT502570 in miRTarBase [35].

Two double-negative feedback motifs between miRNAs and transcription factors were identified in our analysis. The double-negative feedback loop consisting of hsa-miR-183-5p and *ZEB1* has been reported before [86] while the feedback loop consisting of hsa-miR-15a-5p and *E2F7* may be a novel finding. Depending on the strength of regulation, a double-negative feedback loop, which is effectively a positive feedback loop, can bring the cell into a different stable state [95]. In the context of glaucoma, XFG and fibrosis, especially the process of EMT is relevant in which *ZEB1* plays an important role [87]. Apart from hsa-miR-183-5p, also the miR-200 family has been reported to regulate *ZEB1* [96] with a double-negative feedback motif. This interaction did not show up in our results because of the higher FDR of hsa-miR-200c-3p although the p-value of this miR is p = 0.03.

A large proportion of the interactions found in our analysis originates from experiment-based databases and is therefore already validated to some extent albeit usually not in trabecular meshwork cells. Several publications do cover part of our interactome and therefore may serve as validation. In agreement with our interactome, regulation of *ELN*, *COL5A2*, *FSTL1* and *COL1A1* was demonstrated experimentally using a miR-29b mimic and antagomir in primary human skin fibroblasts and other models in the context of treatment of keloid formation and fibroplasia using miR-29b mimic Remlarsen [97]. Further validation of our interactome is provided by another experiment in which human trabecular meshwork cells were transfected with hsa-miR-29b-3p [23] which demonstrated regulation of *ADAM12*, *COL5A1*, *COL1A1* and *SPARC* by this miRNA. Another publication that may serve as validation of our interactome is provided by a similar *in vitro* experiment involving hsa-miR-204-5p which demonstrated regulation of *IL11*, *SERPINE1*, *CXCL3*, *AP1S2*, *BIRC2*, *SERP1*, *SERPINE1*, *PLAUR* by this miRNA in TM tissue [76].

Our *in silico* analysis combines miRNA-Seq and mRNA-Seq (gene) data of the same samples together with putative interactions obtained from experimental and predicting miRNA-mRNA interaction databases to arrive at an interactome that describes how genes in TM cells may be regulated by miRNAs in response to TGF-β1 treatment. Many interactions in the interactome have already been described in other publications which shows the predictive value of our interactome. Despite this fact, there are certain limitations to this study.

The experimental interaction information available in interaction databases is usually not determined in TM tissue while it is known that miRNA regulatory strength of genes has cell type and cell context specific characteristics [47,98].

Our initial interactome was Boolean by nature, i.e. an interaction between miRNA is reported to be either present or not present, but there was no indication of the strength of interaction. In an attempt to predict the strength of interaction beyond just a Boolean yes/no we developed the interaction score which attempts to give an indication of the strength of regulatory action of a certain miRNA-gene pair. One parameter in this score is the molarity of the miRNA (in the form of log_2_CPM), from miRNA-target reaction equations one would expect that the magnitude of differential mRNA expression due to miRNA regulation would depend on the (local) concentration of the regulating miRNA and the binding strength between both species [99,100].

Another parameter which may be a measure of miRNA regulatory strength is correlation between regulating miRNA and regulated mRNA may be calculated [24] and indeed if the gene is regulated by one dominant regulatory miRNA this interaction is likely to show the best (closest to -1.0) correlation but by nature of miRNA regulation, a gene is usually regulated by the combined effort of multiple miRNAs [98]. If these miRNAs have similar regulatory strength and are uncorrelated amongst themselves, then this can result in weaker (i.e. closer to 0.0) correlation values.

Besides miRNAs, also other regulators act on mRNA expression such as lncRNAs and transcription factors which have not been taken into account in this analysis. It is possible that a gene is regulated in one direction by one type of regulator and in the opposite direction by a miRNA. If this is the case, then this miRNA regulation may be missed.

The local concentration of miRNA and mRNA species may differ from what would be expected from the number of reads obtained from RNA-Seq because both miRNA-Seq and mRNA-Seq are bulk sequencing procedures which show the averaged expression of multiple cell-types while the TM is known to consist of several cell-types [101]. This unknown compartmentalisation may therefore lead to false positives if the supposedly interacting miRNA and mRNA in reality reside in different compartments or cell types. To partially solve this problem, it would be better to use single-cell sequencing.

**Fig 8:**
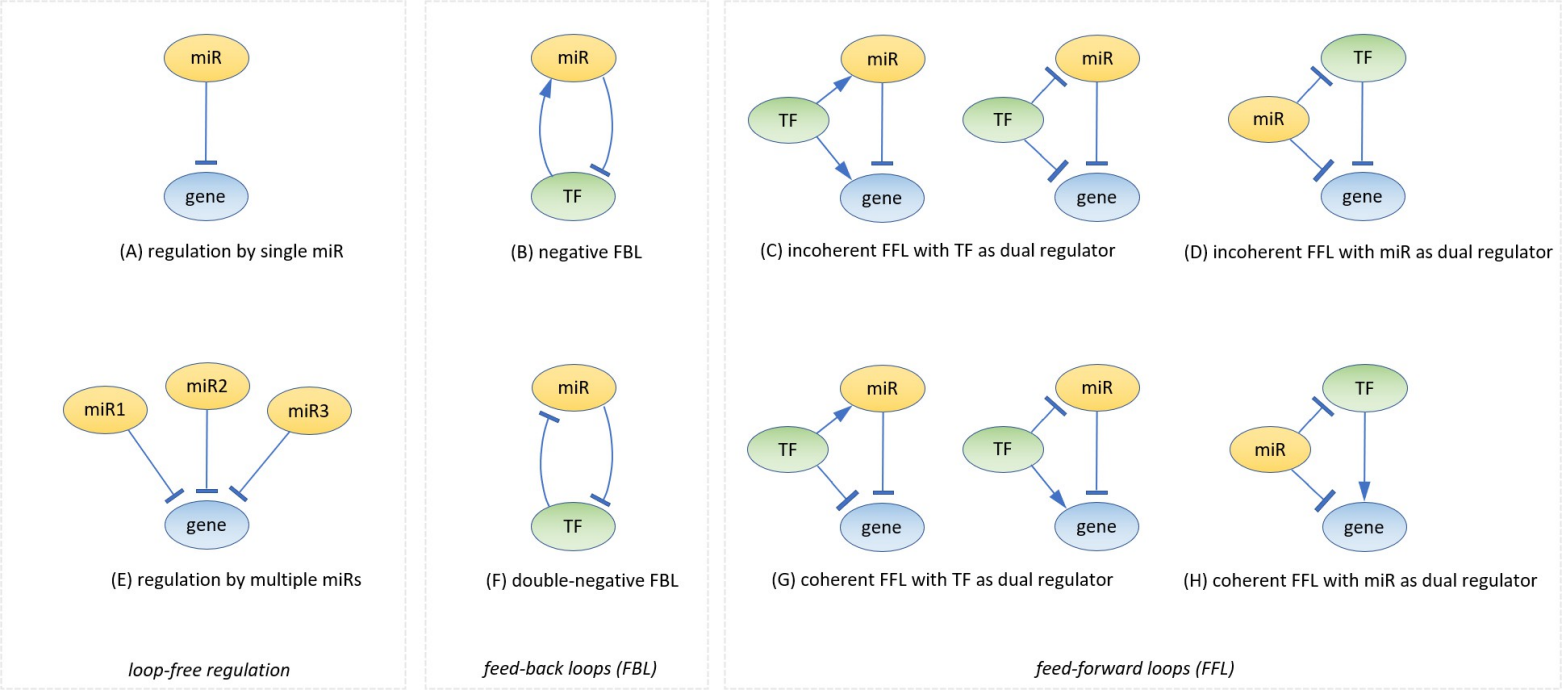
Overview of miRNA regulatory motifs. In which miR = miRNA, gene = mRNA, TF = transcription factor. (A) Basic miRNA-gene interaction (B) negative feedback loop (C) incoherent feedforward loop with TF as dual regulator of both miRNA and regulated gene (D) incoherent feedforward loop with miRNA as dual regulator of both TF and regulated gene (E) regulation of one gene by multiple miRNAs (F) double-negative feedback loop (G) coherent feedforward loop with TF as dual regulator (H) coherent feedforward loop with miRNA as dual regulator. Adapted from [102].

MiRNAs can exert their regulatory role as part of several different regulatory motifs [102], an overview is shown in Fig 8; motif A shows the basic regulatory motif. From this motif, it follows that when the miR expression increases, the target expression should decrease which is the rationale behind applying the constraint of opposite expression as we have done. This simple motif may be valid in case there is one dominant regulating miR or group of miRs in concert such as several interactions involving members of the miR-29 family. A target mRNA may be regulated by miRs of approximately equal regulatory strength (motif E) which may be partially missed by our method if the regulating miRs have unequal signs of expression (some up, some down). Other motifs that will be missed by our method are (B) negative feed-back loop and both incoherent feed-forward loops (C, D) because in these cases the sign of expression of miR and target may be the same. Motifs that will usually be detected by our method are (F) double-negative feedback-loop, and both coherent feed-forward loops (G, H). To complicate things further, a certain regulated target mRNA may be part of multiple different motifs leading to a very complex network indeed of which the interaction parameters are often unknown or inaccurate.

Rather than trying to accurately model the complete interaction network it is also possible to approximate the network by determining dominant regulators (both miRNAs and transcription factors) that regulate groups of co-regulated mRNA species. What mRNA species (genes) are co-regulated can be determined by using correlation-based techniques such as weighted gene correlation network analysis (WGCNA) [103]. To establish by what common miRNA regulators the co-regulated genes are controlled, our Boolean interactome may be employed. A similar methodology has been applied in the context of idiopathic pulmonary fibrosis [103]. Unfortunately, WGCNA requires at least 15 samples [104] so this method could not be applied to our experiment.

## Conclusions

Pseudoexfoliation glaucoma (XFG) shows changes in both mRNA and miRNA expression. When normal HTM tissue is treated with TGF-β1 there is significant overlap in differential mRNA and miRNA expression in this simple model and XFG, but it is often unclear what the regulatory effect of differentially expressed miRNA may be. In this work we present a bioinformatics analysis that provides a hypothesis-generating prediction of the regulatory functions of differentially expressed miRNAs causing differential gene expression in normal HTM tissue when treated with TGF-β1. To our knowledge this is the first study that combines genome-wide miRNA and mRNA expression into one genome-wide interactome for normal TM cells treated with TGF-β1. This interactome may help to understand the pathophysiology of XFG and could be used to identify potential candidates for miRNA-based therapeutics.

## Supporting information

S1 Fig miRNA-gene interactome of genes involved in the Unfolded Protein Response (UPR) for human trabecular meshwork cells stimulated by TGF-β1. Heatmap of the interactome in which the heatmap colour maps to interaction score which is calculated from database evidence, miRNA expression and miRNA-gene correlation using Eq (2).
(TIF)

S2 Fig miRNA-gene interactome of transcription factors for human trabecular meshwork cells stimulated by TGF-β1. Heatmap of the interactome in which the heatmap colour maps to interaction score which is calculated from database evidence, miRNA expression and miRNA-gene correlation using Eq (2).
(TIF)

S1 Table miRNA regulation of biological processes altered by TGF-β1 in HTM cells. This table contains altered biological processes, the genes within these processes and what miRNAs may be regulating these genes.
(XLSX)

S2 Table miRNA-gene interactome for TGF-β1 stimulated HTM cells. This file contains all miRNA-gene interactions.
(XLSX)

S3 Table miRNA-TF loops for TGF-β1 stimulated HTM cells. This file contains miRNA-TF loops.
(XLSX)
