# Supplementary figures and images for: Investigating the miRNA-mRNA interactome of human trabecular meshwork cells treated with TGF-β1 provides insights into the pathogenesis of pseudoexfoliation glaucoma
Source: PLoS One. 2025 Jan 30;20(1):e0318125. doi: 10.1371/journal.pone.0318125 (PMC11781692; doi:10.1371/journal.pone.0318125)

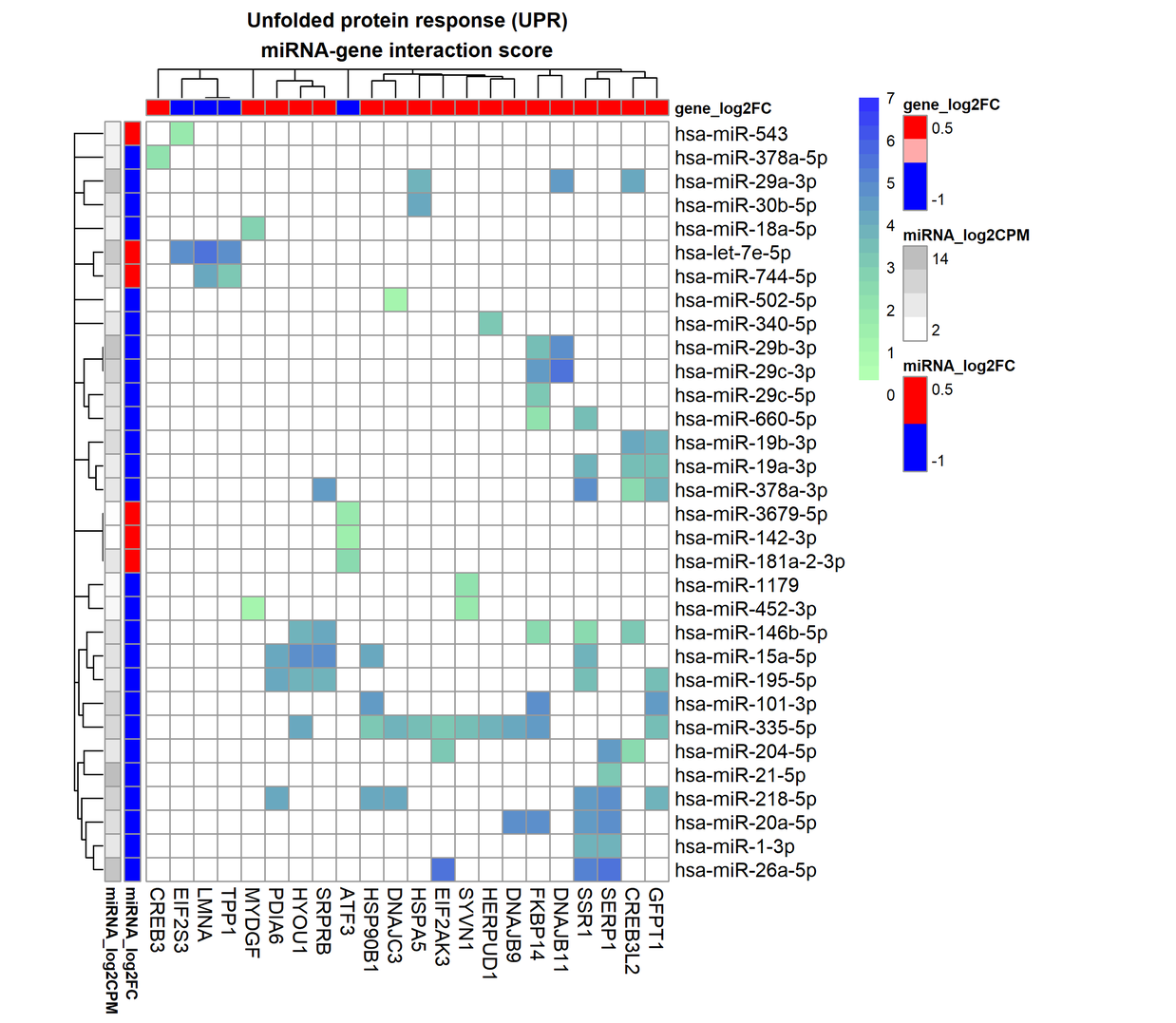

Supplement: S1 Fig — Heatmap of the interactome in which the heatmap colour maps to interaction score which is calculated from database evidence, miRNA expression and miRNA-gene correlation using Eq (2). (TIF) [file pone.0318125.s001.tif]

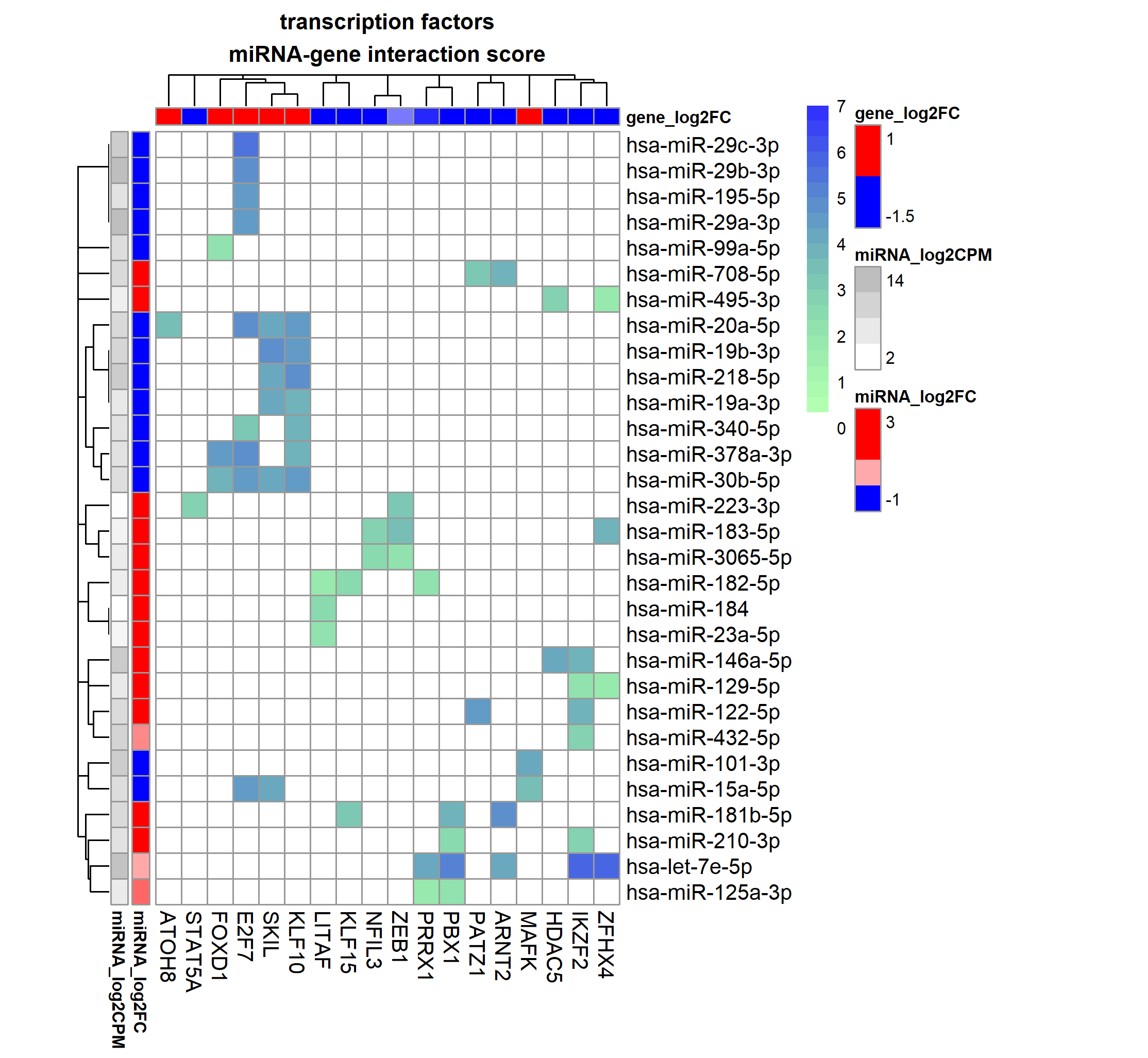

Supplement: S2 Fig — Heatmap of the interactome in which the heatmap colour maps to interaction score which is calculated from database evidence, miRNA expression and miRNA-gene correlation using Eq (2). (TIF) [file pone.0318125.s002.tif]
